# LncRNA GLCC1 promotes colorectal carcinogenesis and glucose metabolism by stabilizing c-Myc

**DOI:** 10.1038/s41467-019-11447-8

**Published:** 2019-08-02

**Authors:** Jiayin Tang, Tingting Yan, Yujie Bao, Chaoqin Shen, Chenyang Yu, Xiaoqiang Zhu, Xianglong Tian, Fangfang Guo, Qian Liang, Qiang Liu, Ming Zhong, Jinxian Chen, Zhizheng Ge, Xiaobo Li, Xiaoyu Chen, Yun Cui, Yingxuan Chen, Weiping Zou, Haoyan Chen, Jie Hong, Jing-Yuan Fang

**Affiliations:** 1 State Key Laboratory for Oncogenes and Related Genes, Key Laboratory of Gastroenterology & Hepatology, Ministry of Health, Division of Gastroenterology and Hepatology, Shanghai Institute of Digestive Disease, Renji Hospital, School of Medicine, Shanghai Jiao Tong University, 145 Middle Shandong Road, 200001 Shanghai, China; 2 Department of Gastrointestinal Surgery, Renji Hospital, School of Medicine, Shanghai Jiao Tong University, 200127 Shanghai, China; 3 Department of Infectious Disease, Shanghai Ninth People’s Hospital, Shanghai Jiao Tong University School of Medicine, Shanghai, 210999 China; 4 Department of Pathology, Renji Hospital, Shanghai Jiao Tong University School of Medicine, Shanghai, 200127 China; 5 Department of Surgery, The University of Michigan Comprehensive Cancer Center, Graduate programs in Immunology and Cancer Biology, University of Michigan School of Medicine, Ann Arbor, MI 48109 USA

**Keywords:** Cancer metabolism, Gastrointestinal cancer

## Abstract

Long non-coding RNAs (lncRNAs) contribute to colorectal cancer (CRC). However, the role of lncRNAs in CRC metabolism, especially glucose metabolism remains largely unknown. In this study, we identify a lncRNA, GLCC1, which is significantly upregulated under glucose starvation in CRC cells, supporting cell survival and proliferation by enhancing glycolysis. Mechanistically, GLCC1 stabilizes c-Myc transcriptional factor from ubiquitination by direct interaction with HSP90 chaperon and further specifies the transcriptional modification pattern on c-Myc target genes, such as *LDHA*, consequently reprogram glycolytic metabolism for CRC proliferation. Clinically, GLCC1 is associated with tumorigenesis, tumor size and predicts poor prognosis. Thus, GLCC1 is mechanistically, functionally, and clinically oncogenic in colorectal cancer. Targeting GLCC1 and its pathway may be meaningful for treating patients with colorectal cancer.

## Abstract (web summary)

lncRNA and cellular metabolism are frequently dysregulated in cancer. In this study, the authors discover the lncGLCC1 is increased in colorectal cancer cells under glucose starvation conditions and correlates with poor prognosis in this cancer.

## Introduction

The incidence and mortality of colorectal cancer (CRC) in adults over the age of 50 have been declining over the past 30 years^1^. However, the incidence and mortality of CRC are rising among young adults^2^. Patients with advanced colorectal cancer have a poor prognosis^3^. Pathological classification is used to assess prognosis and inform the treatment of colorectal cancer. Massive efforts have been made to develop the noninvasive biomarkers to detect early cancer and/or reflect an individual’s cancer risk, which is essential to reduce CRC mortality^4–6^. However, there has been achieved little progress in improving the disease-free survival rate of CRC patients. Since the pathological mechanisms of colorectal cancer progression are not fully understood, more research is needed to discover and develop effective biomarkers and targets for colorectal cancer diagnosis and treatment.

Alteration of energy metabolism, especially abnormal activation of glycolysis pathway in cancer cells is recently recognized as a hallmark of cancer^7^. Lots of cancer cells display activation of glycolysis with more production of lactic acid, which is exported to the microenvironment, leading to a decrease in extracellular pH during glycolytic metabolism^8^. High rate of glycolysis and low pH value in the microenvironment has been associated with poor prognosis in CRC patients^9,10^, and with increased malignant features, including cancer proliferation and metastasis^11,12^.

Long non-coding RNAs (lncRNAs) are a class of non-coding transcripts >200 nucleotides in length. Recent studies have revealed that lncRNAs may affect cancer progression^13,14^. For example, lncRNAs, HOTAIR, MEG3, MALAT-1, H19, and GClnc1 may play a role in carcinogenesis^15–19^. In addition, lincRNA-p21 has been identified as an important player in the regulation of the Warburg effect in carcinogenesis^20^. LncRNA BCAR4 was uncovered as a downstream target of YAP in breast cancer glycolysis progression^21^. The role of lncRNAs and the underlying mechanisms in colorectal cancer has been reported before^15,22,23^. However, more specific mechanisms of lncRNAs in the initiation and glucose metabolism of colorectal cancer need to be further teased out.

Since the fact that glucose supplement and extracellular glucose concentration in tumor tissues are much lower than surrounding normal tissues^24^ has been uncovered, it is critical for cancer cells to survive and proliferate by reprogramming glucose metabolism^25^. In order to find out underlying oncogenic lncRNAs in CRC, we examined genome-wide lncRNA expression profiles in colorectal cancer and paired-noncancerous tissues. Meanwhile, we simulated glucose-limited conditions in vitro for seeking glycolysis-associated lncRNAs. From all the candidate lncRNAs, the lncRNA (AF339830) has been identified and characterized, which is associated with the poor prognosis in colorectal cancer patients, and therefore designated as glycolysis-associated lncRNA of colorectal cancer (GLCC1).

Mechanistically, we find GLCC1 exerts its effect on glycolysis and proliferation indispensable of c-Myc. c-Myc has been extensively documented to regulate glucose metabolism as a critical oncogene in metabolism reprogramming, which makes it to be a key switch in on/off metabolic activity in cancer cells. Besides, many of c-Myc target genes are revealed to be essential for cell growth and cancer progression. Here, we report that GLCC1 stabilizes c-Myc from ubiquitination degradation in cytoplasm by combining with HSP90 (HSP90AA1) chaperon and consequently specifies the pattern of transcription on its target genes, especially lactate dehydrogenase (LDHA), which is a crucial limiting enzyme for catalysis of glycolysis. GLCC1-c-Myc-LDHA, as a cascade reaction orchestrated by GLCC1 under glucose starvation, may be a promising metabolism blocker target for antitumor therapy.

## Results

### GLCC1 is clinically relevant in response to energy stress

**Fig. 1:**
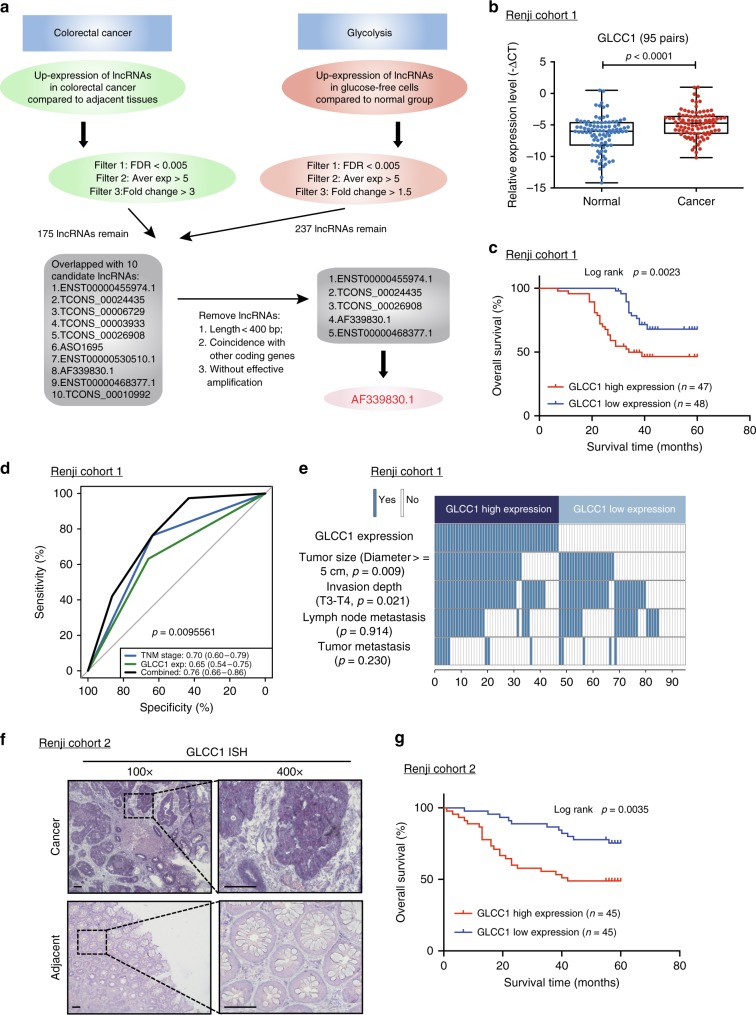
LncRNA candidate AF339830 is clinically relevant in colorectal cancer. **a** The flow chart for selected candidate lncRNAs in upregulated lncRNAs in colorectal cancer tissue and glycolysis-free cells is shown. **b** Statistical analysis of GLCC1 expression in 95 pairs of colorectal cancer and normal tissues, paired *t-*test. In boxplots (middle line depicts the median and the whiskers the min to max. range). **c** Survival was analyzed and compared between patients with low and high levels of GLCC1 in 95 patients with colorectal cancer (cohort 1), log-rank test. **d** ROC analysis of lncRNA GLCC1 in Renji cohort 1. **e** Comparing different tumor size and TNM stage between GLCC1 high-expression and GLCC1 low-expression tumors of Renji cohort 1. The heatmap illustrates the association of different clinical characters with GLCC1 high and low-expression tumors. Statistical significance was performed by the *χ*^2^ test. **f** Representative images of GLCC1 expression in colorectal cancer and adjacent colorectal tissues using ISH analysis in Renji cohort 2. The purple staining represents positive signal. Scale bar indicates 100 μm. **g** Survival was analyzed and compared between patients with high and low levels of GLCC1 expression in tumor in Renji cohort 2; *n* = 90, log-rank test

Two high-through microarrays were designed to probe the specific mechanisms of lncRNAs in colorectal cancer initiation and glucose metabolism. Briefly, The Agilent human lncRNA+mRNA Array V4.0 (4 × 180K format) was used to profile the lncRNA expression in five paired colorectal cancer tissues and adjacent tissues. In total, 175 lncRNA were selected with more stringent filtering criteria (FDR < 0.005, Fold change > 3, Average expression > 5, Fig. 1a). To elucidate glycolysis-related lncRNA in colorectal cancer tumorigenesis, a microarray analysis was performed to compare the gene expression profiles of glucose-free cells and control. A total of 16,768 downregulated genes and 41,234 upregulated genes (FDR < 0.005, Fold change > 1.5, Average expression > 5, Fig. 1a) were detected after glucose starving in colorectal cancer cells. In order to study the glycolysis-related lncRNA, which is highly expressed in colorectal cancer cells, we overlapped these two high-throughput analysis data in our investigation. To screen more functional lncRNA conveniently, more stringent filtering criteria (lncRNA length >400 bp, non-totally overlapped with other coding transcripts, efficient amplification in PCR reaction assay, Fig. 1a) were used in our investigation. Five candidate lncRNAs were finally found and significantly overexpressed in colorectal cancer tissues, which may regulate glycolysis and tumorigenesis as well (Supplementary Data 1).

To validate microarray data, we analyzed the five candidate lncRNAs expression in 95 cases of fresh colorectal cancer and adjacent tissues (Cohort 1, Fig. 1b, Supplementary Fig. 1a–d, and Supplementary data 2). Real-time PCR revealed that two of the five lncRNAs were significantly increased in cancer versus adjacent tissues of cohort 1 (Fig. 1b and Supplementary Fig. 1b). These data indicate that a set of lncRNAs is aberrantly expressed in colorectal cancer tissues.

We next analyzed the correlation between the two lncRNA candidates with the clinical outcome in cohort 1. The Kaplan–Meier analyses showed that lncRNA candidate, lncRNA TCONS_00024435 has no predictive value for the clinical outcome of colorectal cancer patients while high-expression of lncRNA candidate, AF339830, was significantly associated with a poor prognosis in these patients (Supplementary Fig. 1e and Fig. 1c).

To evaluate the pathological and clinical value of lncRNA AF339830, receiver operating characteristic curve (ROC) analysis showed that the area under curve (AUC) of the combination of AF339830-based prediction and TNM-based model (0.76) was higher than the TNM-based model alone (0.70) (Fig. 1d). The data indicate that the combination of AF339830 and TNM stage is more precise in predicting clinical outcome than TNM stage alone. Therefore, we focused our research on AF339830, and henceforth named this lncRNA candidate as glycolysis-associated lncRNA of colorectal cancer (GLCC1).

We next evaluated and compared GLCC1 expression with different clinicopathological features in cohort 1. We found that the GLCC1 expression positively correlated with tumor size and invasion depth (Fig. 1e and Supplementary Fig. 1f). Univariate and multivariate regression analyses of cohort 1 demonstrated that GLCC1 expression was an independent predictor of colorectal cancer aggressiveness with significant hazard ratios for predicting clinical outcome. Its predictive value was comparable to that of the TNM stage (Supplementary Fig. 1g, h).

To further validate the pathological and clinical significance of GLCC1 expression in colorectal cancer, we detected and compared GLCC1 expression by in situ hybridization (ISH) in an additional 90 paraffin-embedded colorectal cancer and adjacent tissues (cohort 2) (Supplementary data 3). GLCC1 expression was higher in colorectal cancer tissues than adjacent tissues (Fig. 1f and Supplementary Fig. 1i). Consistent with the results in cohort 1, high levels of GLCC1 expression were significantly associated with shorter survival time (Fig. 1g) revealed by univariate and multivariate cox regression analyses (Supplementary Fig. 1j, k).

In order to determine whether GLCC1 is a lncRNA, we analyzed its sequence. Northern blot revealed that the size of GLCC1 was ~650 bp in length in colorectal cancer cell lines (Supplementary Fig. 1l). The 5′ and 3′ rapid amplification of complementary DNA (cDNA) ends (RACE)-PCR (Supplementary Fig. 1m) were performed to identify the 5′, 3′ ends, and the transcription start site (TSS) of GLCC1. Sequencing of PCR products revealed the boundary between the universal anchor primer and GLCC1 (Supplementary Fig. 1n). Furthermore, we confirmed that GLCC1 was unlikely to encode any protein product by in vitro translation analysis, indicating that it was a non-coding RNA (Supplementary Fig. 1o). We also have separated the nuclear and cytoplasm fractions in colorectal cancer cell DLD-1 and performed real-time PCR. We found that GLCC1 was mainly located in the cytoplasm (Supplementary Fig. 1p). Collectively, GLCC1 is a lncRNA and highly expressed in colorectal cancer tissues.

### GLCC1 is an oncogenic lncRNA in colorectal cancer

**Fig. 2:**
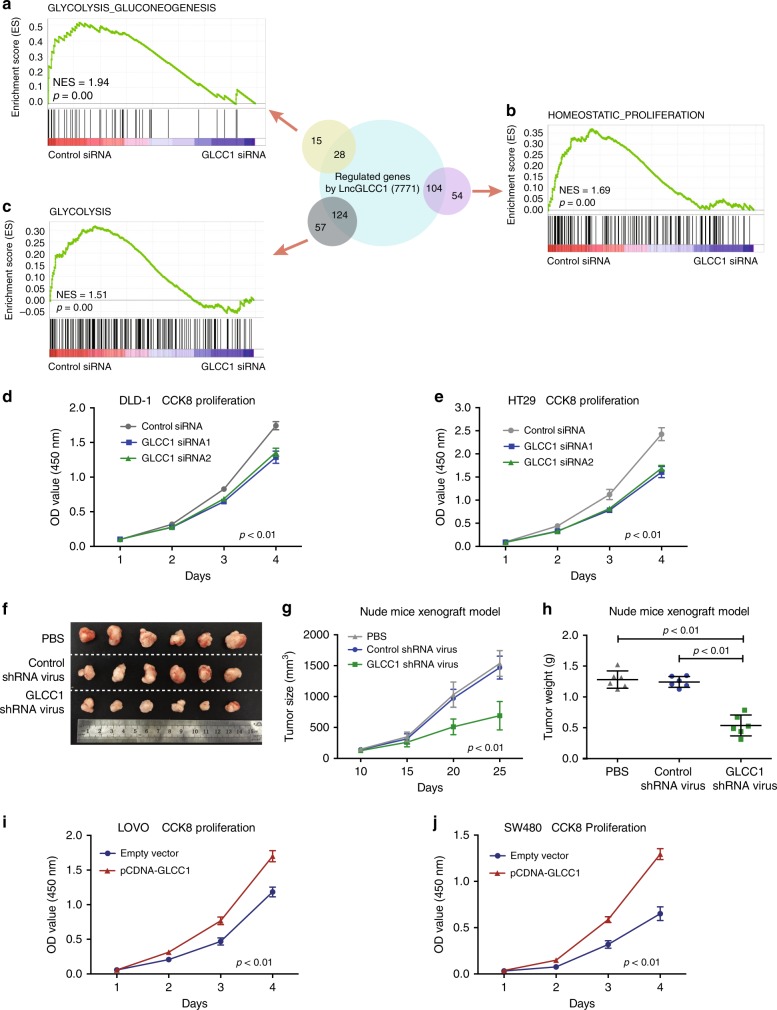
GLCC1 is an oncogenic lncRNA in colorectal cancer. **a**–**c** Overview of GSEA used to identify the differential gene profiles between GLCC1 siRNA transfection colorectal cancer cells and controls. **d**, **e** Cell proliferation assay was performed in DLD-1 and HT29 cells transfected with GLCC1 siRNA1/2; *n* = 6. **f** Representative data of tumors in nude mice-bearing colorectal cancer cells transfected with PBS, control shRNA adenovirus, and GLCC1 shRNA adenovirus; *n* = 6. **g** Tumor volume was measured after GLCC1 shRNA adenovirus treatments in the xenograft mouse model; *n* = 6; *p* < 0.01. **h** Tumor weight was measured in mice after different treatments; *n* = 6. **i**, **j** Cell proliferation assay was performed in LoVo (**i**) and SW480 (**j**) cells transfected with GLCC1 overexpression plasmid; *n* = 3. Data in **d**, **g**, **h**–**j** are presented as the mean ± SE. *p*-values were calculated by one-way ANOVA followed by SNK multiple comparison test

To elucidate whether GLCC1 plays a role in colorectal cancer tumorigenesis, a RNA-seq analysis was performed to compare the gene expression profiles of GLCC1 short-interfering RNA (siRNA) and control siRNA transfectants. A total of 7771 regulated genes (≥2-fold) were detected (raw data accessible via GSE119866) after knockdown of GLCC1 in colorectal cancer cells (Supplementary Data 4). Gene set enrichment analysis (GSEA) revealed that the gene sets related to Glycolysis_Gluconeogenesis (Glycolysis), Glycolysis, and Homeostatic_proliferation (colorectal cancer-specific signature) negatively correlated with GLCC1 downregulation in colorectal cancer cells (Fig. 2a–c). The top-scoring genes recurring in the three gene sets included key glycolysis-related genes, *LDHA* and *G6PD*. Real-time PCR confirmed that alteration of GLCC1 expression dramatically affected the key tumorigenesis and glycolysis gene signatures (Supplementary Fig. 2a). To further gain insight into the biological pathways involved in colorectal cancer pathogenesis, based on the median of GLCC1 expression levels, we performed the GSEA analysis in an independent public dataset from Gene Expression Omnibus (GSE31737) (Supplementary Fig. 2b). Enrichment plots of GSEA showed that the gene signatures of cell proliferation and glycolysis pathways were enriched in patients with high GLCC1 expression, but not in patients with low GLCC1 expression (Supplementary Fig. 2b). These data suggest that GLCC1 may be a key modulator in colorectal tumorigenesis.

To functionally validate the pathway findings, we transfected GLCC1 siRNAs into the colorectal cancer cell lines, DLD-1 and HT29. The two cell lines expressed higher levels of GLCC1 (Supplementary Fig. 2c–e). The results showed that knockdown of GLCC1, significantly impaired colorectal cancer cell proliferation and in DLD-1 cells and HT29 cells (Fig. 2d, e). Knockdown of GLCC1 dramatically reduced DLD-1 tumor growth (Fig. 2f, g) and tumor weight (Fig. 2h) in xenograft mouse tumor models. In support of the pro-tumor role of GLCC1, Ki67 staining revealed that downregulation of GLCC1 decreased tumor cell proliferation in vivo (Supplementary Fig. 2f). In the gain-of-function assays, overexpression of GLCC1 increased cell proliferation of LoVo and SW480 cells in vitro (Supplementary Fig. 2g, h and Fig. 2i, j). These data strongly suggest that GLCC1 may promote colorectal cancer progression by regulating colorectal cancer cell proliferation.

### GLCC1-induced cell survival depends on glycolytic metabolism

**Fig. 3:**
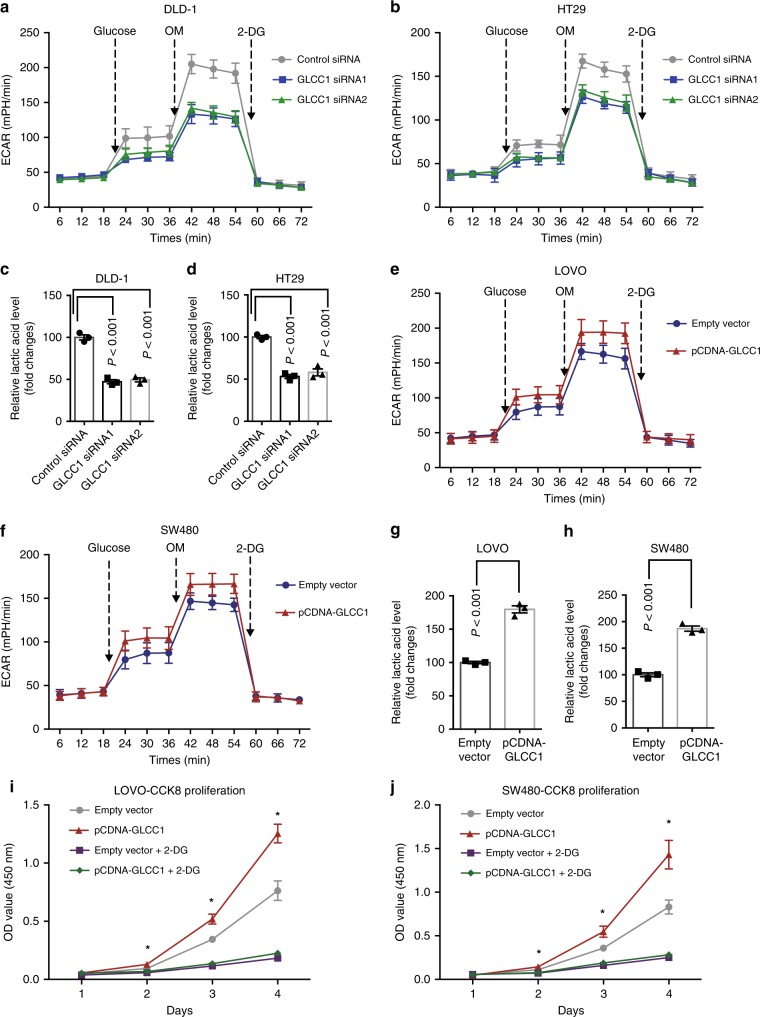
GLCC1-induced cell survival depends on glycolytic metabolism. **a**, **b** Extracellular acid ratio (ECAR) upon cells were measured after transfecting with control or GLCC1 siRNA1/2 in DLD-1 (**a**) and HT29 (**b**). OM, oligomycin; 2-DG, 2-deoxyglucose. **c**, **d** The relative lactic acid level was detected in DLD-1 (**c**) and HT29 (**d**) cells transfected with control or GLCC1 siRNA1/2. **e**, **f** The change of ECAR level with different treatment in LoVo (**e**) and SW480 (**f**) cells was measured after transfecting with control or GLCC1 overexpression plasmid. **g**, **h** The relative lactic acid level in LoVo (**g**) and SW480 (**h**) cells was examined transfected with control or GLCC1 overexpression plasmid. **i**, **j** Cell proliferation assay was performed in LoVo and SW480 cells transfected with control plasmid, GLCC1 overexpression plasmid, 2-DG, 2-DG and GLCC1 overexpression plasmid (*n* = 6). Data are presented as the mean ± SE. *p*-values were calculated by one-way ANOVA followed by SNK multiple comparison test. *p < 0.05

We next tested if altered GLCC1 levels directly influence glycolytic metabolism in colorectal cancer cells by measuring extracellular acidification rate (ECAR)^26^. Indeed, knockdown of GLCC1 significantly reduced ECAR levels in DLD-1 (Fig. 3a) and HT29 cells (Fig. 3b), compared to control cells. In addition, we measured production of extracellular lactic acid, a key metabolite of glycolysis. As shown in Fig. 3c, lactic acid production was significantly decreased after GLCC1 downregulation in DLD-1 (Fig. 3c) and HT29 cells (Fig. 3d). In-gain-of function assays, overexpression of GLCC1 dramatically increased ECAR levels (Fig. 3e, f), as well as lactic acid production (Fig. 3g, h) in LoVo and SW480 cells. In addition, 2-DG (an inhibitor of glycolysis pathway) treatment significantly blocked GLCC1-induced cell proliferation and lactic acid production in LoVo (Fig. 3i and Supplementary Fig. 3a) and SW480 cells (Fig. 3j and Supplementary Fig. 3b). Furthermore, glucose starvation significantly increased GLCC1 expression in DLD-1 cells (Supplementary Fig. 3c). These data indicate that GLCC1 may be a glucose starvation-induced lncRNA and regulate glycolytic metabolism in colorectal cancer.

### GLCC1 interacts with HSP90 and regulates c-Myc stability

**Fig. 4:**
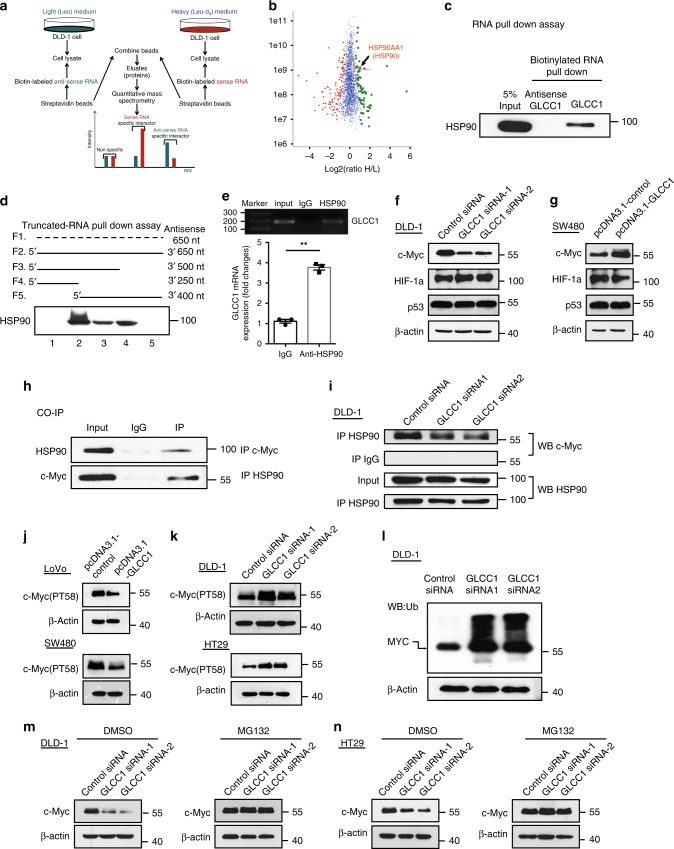
GLCC1 interacts with HSP90 and regulate the stability of c-Myc. **a** Schematic design of using SILAC-based quantitative proteomic approach to identify the GLCC1-specific interactors. **b** Proteome-wide accurate quantification and significance. The logarithm of normalized protein ratios (*H*/*L* ratio) are plotted against protein intensities. The filled green circles represent the sense RNA-specific interactors (*p* < 0.05, 74 proteins, *H*/*L* ratio > 1.46), the red marked one is the validated sense-RNA interactor HSP90. The filled red diamonds represent the anti-sense RNA-specific-binding partners ((*p* < 0.05, 98 proteins, *H*/*L* ratio <0.66). As the anti-sense RNA is an “artificial” RNA, these proteins can be considered as non-specific binding. The blue crosses represent all the non-specific-binding proteins (*p* > 0.05, 1964 proteins). **c** Western blot of the proteins from anti-sense GLCC1 and GLCC1 pull-down assays; **d** Western blot of HSP90 in samples pulled down by full-length (F2) or truncated GLCC1 (F3: 1–500, F4: 1–250, F5: 1251–650); **e** RNA immunoprecipitation experiments were performed using anti-HSP90 antibody, and specific primers were used to detect GLCC1. **f** Western blot of the proteins from control and GLCC1 siRNA transfection DLD-1 cells. **g** Western blot of the proteins from control and GLCC1 overexpression plasmid SW480 cells. **h** Co-immunoprecipitation detected the interaction of HSP90 and c-Myc in the DLD-1 cells. **i** Immunoprecipitation assay was performed to detect the interaction between HSP90 and c-Myc after transfection of GLCC1 siRNA. **j** Western blot of c-Myc (PT56) expression in control or GLCC1 overexpression sample in SW480 and LoVo cells. **k** Western blot of c-Myc (PT56) expression in control or GLCC1 siRNA sample in DLD-1 and HT29 cells. **l** Western blot of the total ubiquitination proteins and c-Myc from control and GLCC1 siRNA1/2 transfection DLD-1 cells. **m**, **n** Western blot of c-Myc from control and GLCC1 siRNA1/2 transfection samples treated with DMSO or ubiquitination inhibitor MG132 in DLD-1 and HT29 cells

To dissect the mechanistic role of the lncRNA in carcinogenesis, we applied SILAC-based proteomic approach^27,28^ in combination with pull-down technique to screen the lncRNA-interacting proteins. As shown in Fig. 4a, In MS analysis, for each leucine-containing peptide, a pair of isotope signals with a mass split of 3*n/z* (*n* represents the number of leucine in the peptide, and *z* is the charge number of the peptide) could be observed; the light isotope peak is originally from anti-sense GLCC1 pulled-down protein and the heavy one comes from sense RNA’s pull-down. Consequently, 74 (*H*/*L* ratio >1.45) and 98 (*H*/*L* ratio <0.66) were, respectively, distinguished as sense and anti-sense-RNA-specific interactors (Fig. 4b). Among the top 15 specific interactors of sense GLCC1 (Supplementary Data 5), we selected those proteins, which may participate in glycolysis (HSP90AA1, HSP90AB1), for further binding validation. Western blot showed that only HSP90 (the former name of HSP90AA1) bound specifically to GLCC1, but not HSP90AB1 (Fig. 4c and Supplementary Fig. 4a). The data suggest that HSP90 may interact with GLCC1. HSP90 is a protein chaperon, which is essential for the stability and function of many proteins, which mediate important biological functions, including cell survival and carcinogenesis^29–31^. To identify the HSP90-interacting region of GLCC1, we constructed and biotinylated four fragments of GLCC1 (F1: full-length of anti-sense GLCC1, F2: full-length of sense GLCC1, F3: 1–500 bp, F4: 1–250 bp, F5 250–650 bp), and used them in the pull-down assay with DLD-1 cell lysates. We found that the 5′ fragment of GLCC1 mediated the interaction with HSP90 (Fig. 4d). To substantiate the observation, anti-HSP90 antibodies were used to immunoprecipitate endogenous HSP90 from cell lysate of DLD-1 cells. To this end, RNAs bound to HSP90 were extracted and analyzed. PCR data revealed that GLCC1 directly bound with HSP90 in colorectal cancer cells (Fig. 4e, upper panel). We also detected ~4-fold enrichments of GLCC1 in the anti-HSP90 immunoprecipitates, compared with the IgG control (Fig. 4e, down panel), and glucose starvation significantly increased the binding efficiency between HSP90 and CLCC1 (Supplementary Fig. 4b). Thus, GLCC1 may specifically bind with HSP90 in colorectal cancer cells and this binding is glucose starving-dependent.

We next constructed pCDNA3.1-HSP90 (∆NTD), pCDNA3.1-HSP90 (∆MD), and pCDNA3.1-HSP90 (∆CTD), with Flag-tag, respectively (Supplementary Fig. 4c). These recombinant plasmids were transfected into DLD-1 CRC cells and successfully overexpressed (Supplementary Fig. 4c). RNA-binding protein immunoprecipitation (RIP) assay was performed later. Real-time PCR data showed that deletion of HSP90 MD domain dramatically decreased the binding efficiency between this truncated protein with lncRNA GLCC1 (Supplementary Fig. 4d). However, this phenomenon was not observed in the mutated HSP90 with NTD or CTD domain deletion, suggesting that the MD domain of HSP90 is essential for this protein binding to GLCC1. In addition, overexpression or downregulation of GLCC1 had no effect on the expression of HSP90 (Supplementary Fig. 4e). Since HSP90 may function as a molecular chaperon and stabilize the transcription factor, protein kinase and oncogenic protein in tumor signaling pathways, we next hypothesize that GLCC1 is responsible for the stability of the complex between HSP90 and its target protein. We next detected the target-interacting proteins of HSP90 in glycolysis progression, including c-Myc, HIF-1α, and P53^32–35^. Western blot showed that knockdown of GLCC1 decreased c-Myc expression, and overexpression of GLCC1 increased c-Myc expression (Fig. 4f, g). While no matter loss of function of GLCC1 or gain of function of GLCC1, had no influence on HIF-1α or P53 (Fig. 4f, g). CO-IP assay further confirmed that HSP90 could specifically bind to c-Myc (Fig. 4h). Furthermore, knockdown of GLCC1 treatment impaired the interaction between HSP90 and c-Myc (Fig. 4i). In vitro validation assay, we purified GST-c-Myc and His-HSP90 fusion proteins to perform the GST pull-down assay with or without GLCC1 lncRNA. Western blot data showed that GST-c-Myc directly interacted with His-HSP90 (Supplementary Fig. 4f). GLCC1 lncRNA may strengthen the interaction between c-Myc and HSP90, indicating the interaction between HSP90 and c-Myc is GLCC1 dose-dependent (Supplementary Fig. 4f). Moreover, 17AAG^36^ (17-N-Allylamino-17-demethoxygeldanamycin, the first HSP90 inhibitor in clinical trials) treatment decreased GLCC1-mediated c-Myc upregulation (Supplementary Fig. 4g), suggesting that GLCC1 is responsible for the interaction between HSP90 and c-Myc and the protein level of c-Myc. As reported, multiple ubiquitin ligases interact and ubiquitinate c-Myc in cytoplasm, thus targeting it for proteasome-mediated degradation^33^. We next found that overexpression of GLCC1 decreased the level of ubiquitin marker of c-Myc (PT58)^37^ (Fig. 4j). Downregulation of GLCC1 increased the level of c-Myc (PT58) and the ubiquitination of c-Myc protein in CRC cells (Fig. 4k). Glucose starving decreased the level of c-Myc (PT58) and increased c-Myc expression in DLD-1 cells (Supplementary Fig. 4h). Furthermore, MG132 treatment blocked GLCC1 siRNA-induced c-Myc degradation in DLD-1 and HT29 (Fig. 4m, n) cells. Since deubiquitinating enzymes USP22 and USP28 may regulate c-Myc expression via modulating its ubiquitylation^38–40^, we next explored whether USP22 and USP28 participate in GLCC1-induced c-Myc stabilization. Western blot data showed that knockdown of USP22 (Supplementary Fig. 4i), but not USP28 (Supplementary Fig. 4j) significantly increased the expression level of PT58-c-Myc, which is the ubiquitination activating marker of c-Myc, and decreased c-Myc protein expression in DLD-1 cells. These data indicate that USP22 specifically regulate the ubiquitination of c-Myc in CRC cells. Further Co-IP western blot assay showed that USP22 could bind with HSP90 (Supplementary Fig. 4k). Downregulating of GLCC1 impaired the interaction between USP22 and HSP90 (Supplementary Fig. 4l), indicating that lncRNA GLCC1 mediates the interaction between USP22 and HSP90 and further modified the ubiquitination and protein levels of c-Myc. Thus, GLCC1 is important for c-Myc and HSP90 interaction, and GLCC1 is responsible for c-Myc protein stability.

### C-Myc participates in the biological function of GLCC1

**Fig. 5:**
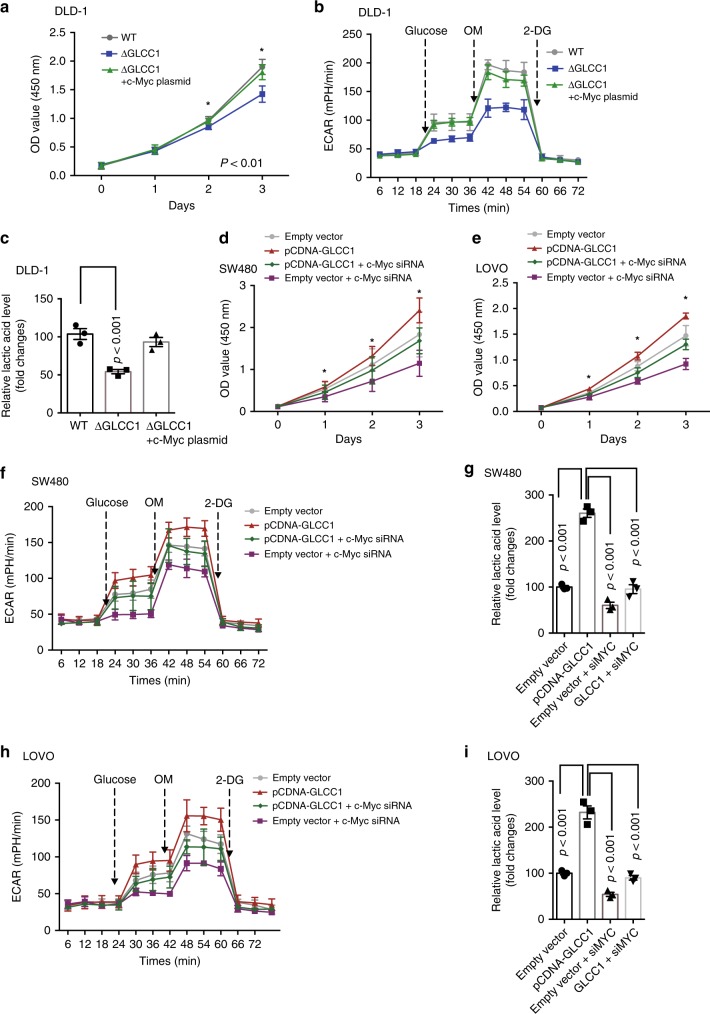
C-Myc participate in the biological function of GLCC1 in CRC cells. **a** Cell proliferation assay was performed in DLD-1-wt, DLD-1ΔGLCC1 cells transfected with control or c-Myc overexpression plasmid; *n* = 6. **b** The change of ECAR level was detected in DLD-1-wt, DLD-1ΔGLCC1 cells transfected with control or c-Myc overexpression plasmid; *n* = 3. **c** The relative lactic acid level was measured in DLD-1-wt, DLD-1ΔGLCC1 cells transfected with control or c-Myc overexpression plasmid; *n* = 3. **d**, **e** Cell proliferation assay was performed in SW480 (**d**) and LoVo (**e**) cells cotransfected with control and c-Myc siRNA or cotransfected with GLCC1 overexpression plasmid and c-Myc siRNA; *n* = 6. **f** The change of ECAR level was detected in SW480 cells cotransfected with control and MYC siRNA or cotransfected with GLCC1 overexpression plasmid and c-Myc siRNA; *n* = 3. **g** The relative lactic acid level was measured in SW480 cells cotransfected with control and c-Myc siRNA or cotransfected with GLCC1 overexpression plasmid and c-Myc siRNA; *n* = 3. **h** The change of ECAR level was detected in LoVo cells cotransfected with control and c-Myc siRNA or cotransfected with GLCC1 overexpression plasmid and c-Myc siRNA; *n* = 3. **i** The relative lactic acid level was measured in LoVo cells cotransfected with control and c-Myc siRNA or cotransfected with GLCC1 overexpression plasmid and c-Myc siRNA; *n* = 3. Data are presented as the mean ± SE. *p*-values were calculated by one-way ANOVA followed by SNK multiple comparison test. *p < 0.05

We next hypothesized that c-Myc mediated the biological function of GLCC1 in colorectal cancer. To test this hypothesis, we used CRISPER-Cas9 system to constructed DLD-1 cells with lncRNA GLCC1 deletion (DLD-1∆GLCC1). The cell proliferation (Fig. 5a), ECAR values (Fig. 5b) and lactate production (Fig. 5c) were significantly reduced in GLCC1-depleted DLD-1 cells, compared with WT DLD-1 cells. Overexpression of c-Myc significantly rescued GLCC1 KO-induced decrease in cell proliferation (Fig. 5a and Supplementary Fig. 5a) and lactate production (Fig. 5b, c). To further confirm the results, we transfected c-Myc siRNA into GLCC1-overexpressed cells and found that knockdown of c-Myc expression significantly reduced GLCC1-induced increase in colorectal cancer cell proliferation in SW480 (Fig. 5d and Supplementary Fig. 5b) and LoVo (Fig. 5e and Supplementary Fig. 5c) cells. In glycolysis metabolism assays, downregulation of c-Myc dramatically blocked GLCC1-induced increase in ECAR levels, as well as lactic acid production in SW480 (Fig. 5f, g) and LoVo cells (Fig. 5h, i). Moreover, Knockdown of c-Myc, 17AAG or 2-DG treatment dramatically reduced GLCC1-induced increased in tumor growth (Supplementary Fig. 5d, f) and tumor weight (Supplementary Fig. 5e) in xenograft mouse tumor models. These data indicate that GLCC1-regulated glycolytic metabolism and cell proliferation dependents on HSP90-mediated c-Myc stability in colorectal cancer.

### GLCC1 co-ordinates the localization of c-Myc genome-wide

**Fig. 6:**
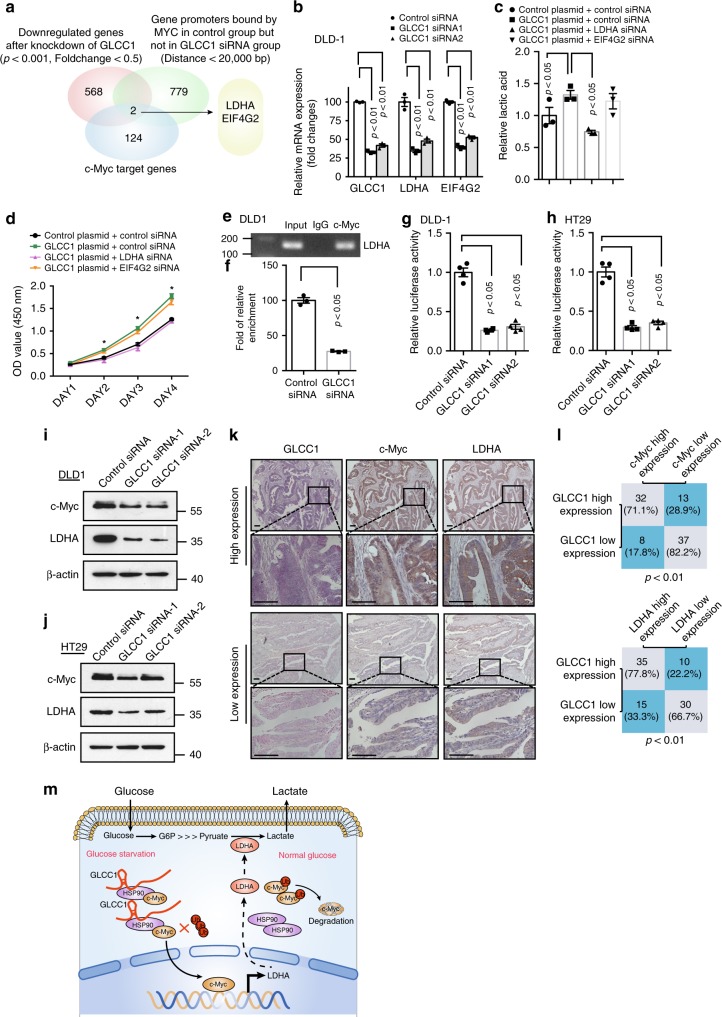
GLCC1 co-ordinates the localization of c-Myc genome-wide. **a **Venn diagram shows the gene promoters occupied by c-Myc in control but not in lncGLCC1 siRNA-transfected cells (Distance <20,000 bp, 779 genes), downregulated after knockdown of lncGLCC1 (*p* < 0.001, Fold change < 0.5, 568 genes), and are target genes of c-Myc (124 genes). **b** Real-time PCR of target genes were performed in DLD-1 cells after transfection of GLCC1 siRNA1/2. **c** Cell supernatant was harvested for lactic acid level detection in DLD-1 cells cotransfected with GLCC1 overexpression plasmid LDHA, EIF4G2, or control siRNA. **d** The OD value at 450 nm was detected in DLD-1 cells cotransfected with GLCC1 overexpression plasmid LDHA, EIF4G2, or control siRNA. **e** LDHA DNA was detected in the chromatin sample immunoprecipitated from DLD-1 cells using an antibody against c-Myc; **f** Real-time PCR of the ChIP samples shows the binding efficiency of c-Myc to the LDHA gene promoter. **g**, **h** Luciferase reporter vectors were generated by inserting the promoter region (–1500 to 0 bp) of the LDHA gene. The reporter vectors were then cotransfected into DLD-1 (**g**) and HT29 (**h**) cells with either GLCC1 siRNA or control siRNA. Cells were harvested for luciferase activity assay. **i**, **j** Western blot of proteins from control or GLCC1 siRNA transfection group were performed in DLD-1 (**i**) and HT29 (**j**). Data in **b**–**h** are presented as the mean ± SE. *p*-values were calculated by one-way ANOVA followed by SNK multiple comparison test. *p < 0.05. **k** The representative images of ISH of GLCC1 and immunohistochemical staining of c-Myc and LDHA in cohort 2. Scale bar indicates 100 μm. **l** Statistical analysis of colorectal cancer tissues under different staining conditions in cohort 2. **m** Schematic representation for the mechanism of GLCC1/HSP90/LDHA axis as a switch that regulates glucose metabolism in human colorectal cancer progression by stabilizing and specifying the transcription modification pattern of c-Myc

To address whether GLCC1 modulates c-Myc genomic binding genome-wide, we performed ChIP coupled with high-throughput sequencing (ChIP-seq) for c-Myc in DLD-1 cells. The binding genes of C-myc were detected in DLD-1 cells (Supplementary Data 6). Further analysis showed only two genes LDHA and EIF4G2, which are the target gene of c-Myc, and are regulated by c-Myc, as well as GLCC1 (Fig. 6a and Supplementary Fig. 6a, b). To further verify these findings, we characterize the relationship between GLCC1 and c-Myc-targeted genes (LDHA and EIF4G2) in DLD-1 CRC cells. We observed that downregulation of GLCC1 significantly reduced the expression of LDHA and EIF4G2 genes in DLD-1 (Fig. 6b) and HT29 cells (Supplementary Fig. 6c). In addition, colorectal cancer cell proliferation and glycolytic metabolism were compared after knockdown LDHA and EIF4G2. However, we found that only downregulation of LDHA, but not EIF4G2, abolished GLCC1-induced cell proliferation and lactic acid production in CRC cells (Fig. 6c, d). This suggests that GLCC1 may bind with HSP90 and stabilize c-Myc protein, thereby controlling its ability to regulate *LDHA* gene expression, and then regulate glycolysis and cell proliferation.

We next explored the mechanism by which GLCC1 regulates the expression of LDHA. Real-time ChIP PCR showed that the binding efficiency of c-Myc on *LDHA* promoter was significantly decreased in DLD-1 (Fig. 6e, f) transfected with GLCC1 siRNAs. We next examined the effect of GLCC1 on the transcriptional activity of *LDHA* gene. Luciferase assay revealed that knockdown of GLCC1 impaired the transcriptional level of *LDHA* promoter in DLD-1 (Fig. 6g) and HT29 cells (Fig. 6h). Western blot data showed that *LDHA* expression was significantly decreased in DLD-1 (Fig. 6i) and HT29 (Fig. 6j) cells transfected with two different GLCC1 siRNAs. The data suggest that GLCC1 may positively regulate *LDHA* transcription in colorectal cancer cells. It has been reported that high levels of *LDHA* expression correlated with poor clinical outcome in colorectal cancer, and this gene may regulate glycolytic metabolism, and then promote cancer cell proliferation and invasion^41,42^.

We next performed immunohistochemical staining in CRC patient’ tissues of cohort 2. Interestingly, the samples with GLCC1 higher expression displayed strongly staining for c-Myc and LDHA (Fig. 6k, upper panel). In addition, samples with low-expression of GLCC1 appeared low levels of c-Myc, LDHA, and HK2 (Fig. 6k, down panel). The data are statistically significant (Fig. 6l). The data indicate that GLCC1 expression is positively correlated with c-Myc and LDHA expression in CRC tissues.

## Discussion

Various oncogenic pathways may contribute to colorectal cancer carcinogenesis^43,44^, however, the potential involvement of lncRNA(s) is poorly defined in human colorectal cancer, as well as in glycolytic metabolism. Through a combination of genomic, biochemical, and cell biological analyses, we have demonstrated that GLCC1 is an oncogenic lncRNA in colorectal cancer. GSEA analyses have demonstrated that cell proliferations, glycolytic pathways in cancer are significantly enriched in response to GLCC1 alteration in the colorectal cancer patients’ datasets. The bioinformatics analyses have been functionally validated in several in vitro and in vivo experimental models. In cultured CRC cells and xenograft mouse models, downregulation of GLCC1 markedly suppresses cell growth and inhibits glycolysis progression in colorectal cancer. The data consistently point to the notion that high GLCC1 expression is a decisive factor of controlling human colorectal cancer aggressiveness.

Cytoplasmic lncRNAs may participate in regulating protein stability and modification^45,46^, however, the underlying molecular mechanisms remain unknown. Our SILIC mass spectrometry and RNA pull-down data have demonstrated that GLCC1 directly interacts with HSP90 chaperon and further stabilizes c-Myc protein, thus increases the transcriptional level of LDHA, then finally activates glycolytic metabolism. This notion is supported by three lines of experimental evidence. (i) GLCC1 directly binds with HSP90 via its 5′ domain; (ii) Genetic deficiency of GLCC1 of the immunopreciptation products abrogates the interaction between HSP90 and c-Myc, and further increases the ubiquitination of c-Myc; (iii) Knockdown of GLCC1 significantly disrupts the binding of the c-Myc to the promoter regions of c-Myc occupied genes. In support of our observation, lncRNAs LINC01138, and lnc-DC, assist stability and modification PRMT5 and p-STAT3, facilitate their protein and enhance their functions^45,46^. Briefly, GLCC1 is a lncRNA capable of modulating the interaction of the HSP90 and c-Myc complex and further altering the binding pattern of c-Myc in promoter regions and transactivating target genes in colorectal cells (Fig. 6m).

When we explored the mechanisms by which GLCC1 contributes to colorectal carcinogenesis, we found the involvement of LDHA. It has been reported that LDHA expression is upregulated in colorectal cancers^47^. This gene may promote glycolysis and is regulated by c-Myc in a transcriptional manner^42^. Consistent with these studies, we show that GLCC1 stabilizes c-Myc protein, and further facilitates this transcription factor to bind to *LDHA* promoter region, then to activate its transcription. In addition, LDHA is functionally responsible for GLCC1-mediated colorectal carcinogenesis and glycolytic metabolism. Notably, as GLCC1 may be directly and indirectly linked to gene regulatory networks, in addition to LDHA, we do not rule out the involvement of other genes in GLCC1-associated biological function.

The cancer cells avidly use the less efficient glycolysis pathway for energy production. This was first described by Otto Warburg in the 1920s, and is also known as the Warburg effect^48^. However, as most of cancer cells are sensitive to glucose deprivation and die eventually under conditions without glucose, one would wonder what the significance of the proposed adaptation in metabolism is for cancer cell survival and progression. Here, we demonstrated that GLCC1 increased under glucose deprivation conditions, solidated c-Myc/LDHA expression, and facilitated glycolysis pathway in CRC cells. The aberrant activation of GLCC1/c-Myc/LDHA glycolysis cascade might be cancer’s comprehensive metabolic adaptation to allow cancer cells to survive from glucose starvation. As a consequence, we inferred that GLCC1 was a potential key lesion to coordinate the metabolic switch for reprogramming under glucose stress. It has been documented that the expression of both c-Myc and its target gene LDHA was dramatically elevated under glucose deprivation condition in hepatocellular carcinoma cell lines, which enlightens us to further explore the reconstitution of glycolysis in CRC cells orchestrated by GLCC1 in our next strategy.

Therefore, from a therapeutic perspective, the mechanistic understanding of glucose metabolism in cellular regulation will enable the identification of therapeutic target. Profiling of lncRNA epigenetic regulation that is affected in glucose metabolism may also allow the development of diagnostic tests of cancer. In short, GLCC1 is the oncogenic lncRNA capable of modulating the interaction between c-Myc and HSP90, and further stabilizing c-Myc protein and regulate glycolytic metabolism via LDHA in colorectal cancer cells (Fig. 6m).

Given the clinical, genetic, biochemical, and functional significance of GLCC1 in colorectal cancer, we conclude that GLCC1 and its associated pathway is crucial for colorectal carcinogenesis as well as glycolysis pathway, and targeting this pathway may be pivotal in the prevention or treatment of colorectal cancer.

## Methods

### Patient specimens

Tumors and the adjacent colorectal tissues were obtained from patients with colorectal cancer who underwent surgery at Shanghai Renji Hospital. The study protocol was approved by the ethics committee of Shanghai Jiao Tong University School of Medicine (Shanghai, China). Written informed consent was obtained from all participants in this study. All the research was carried out in accordance with the provisions of the Declaration of Helsinki of 1975. None of these patients had received radiotherapy or chemotherapy prior to surgery.

### Cell culture and treatment

Human colorectal cancer cell lines SW1116, SW480, Caco2, LoVo, HT29, RKO, DLD-1, and HCT116 were purchased from American Type Culture Collection (ATCC). The cell lines were tested for mycoplasma contamination before used to ensure that they were mycoplasma-free. The small interfering RNAs (siRNAs, 50 nM) against human GLCC1, HSP90, c-Myc, and LDHA were transfected into the colorectal cells using the DharmaFECT 1 siRNA transfection reagent (Thermo Scientific Dharmacon Inc., USA), while non-specific siRNA was used as negative controls. All the siRNAs were purchased from Genepharma Technology (Shanghai, China). The plasmids were transfected into the colorectal cells using the FuGENE transfection reagent (Life Technologies, USA), while non-specific plasmid was used as negative controls. The sequences of the siRNA and plasmids were listed in Supplementary Table 1 and Supplementary Data 7.

### Bioinformatics analysis

Human exon arrays for colorectal cancer and normal adjacent tissues were downloaded from the NCBIs Gene Expression Omnibus (GEO, GSE31737). The datasets GSE31737 consisted of 40 paired colorectal cancer and adjacent tissues. To gain further insight into the biological pathways involved in colorectal cancer pathogenesis through GLCC1, the gene set enrichment analysis (GSEA) was performed. The gene sets showing FDR, 0.25, a well-established cutoff for the identification of biologically relevant genes, were considered enriched between the classes under comparison. The gene sets collection (c2.all.v5.0.symbols.gmt) from the Molecular Signatures Database–MsigDB (http://www.broad.mit.edu/gsea/msigdb/index.jsp) was used for the enrichment analysis. The RNA-seq data can be accessed by GEO Series accession number GSE119866.

### Cell proliferation

Cell proliferation was assessed by the Cell Counting Kit-8 (Dojindo, Japan). Briefly, control and treated colorectal cancer cells were seeded onto 96-well plates at an initial density of 3 × 10^3^ cells per well. CCK-8 (10 μl/well) was added to the cells at specified time points. After incubating for 2 h, the reaction product was quantified according to the manufacturer’s instructions.

### RNA analysis, extraction, and quantitative real-time PCR

The RNA expression levels were measured using a real-time quantitative PCR system. Total RNA was extracted by TRIzol reagent (Invitrogen), and 1 μg of total RNA was reverse-transcribed using the PrimeScriptP RT Reagent Kit (Perfect Real-Time; Takara). The amplified transcript level of each specific gene was normalized to ACTB. The primers (Supplementary Table 2) were provided by Shenggong Company.

### Measurement of ECAR

The Seahorse Extracellular Flux Analyzer XF96 (Seahorse Bioscience) was used to monitor in vitro cells metabolic alternations, according to the manufacturer’s instructions. Cells, transfected with control siRNA, GLCC1 siRNA, control plasmid, and GLCC1 overexpressing plasmid, were seeded in a XF96-well plate at a density of 8 × 10^3^ per well and allowed to attach overnight, followed by serum starvation for 24 h. For detection of the real-time glycolytic rate (ECAR), an indicator of net proton loss during glycolysis, cells were incubated with unbuffered medium followed by a sequential injection of 10 mM glucose, 1 µM oligomycin, and 80 mM 2-deoxyglucose. ECAR measurements was normalized to total protein content and reported as mpH/min. Each sample was determined in triplicate.

### Measurement of lactate

Cells were seeded into six-well plate, transfected with control siRNA, GLCC1 siRNA, control plasmid and GLCC1 overexpressing plasmid. The culture medium was collected for measurement of lactate concentration after cells were starved for 24 h. Lactate production in the medium was detected by using the Lactate Assay Kit (Abcam, l-Lactate Assay kit (colorimetric), CA, USA) according to the manufacturer’s instruction. Results were normalized on the basis of the total protein concentration of each sample. All the experiments were performed in triplicate.

### RNA pull-down assay and mass spectrometry (LC-MS) analysis

Powdered RPMI Media for SILAC (88426) was purchased from Thermo Scientific; Deuterium-labeled leucine (5,5,5-d_3_ (Leu-d_3_), DLM-1259) was obtained from Cambridge Isotope Laboratories (CIL); Dialyzed Fetal Bovine Serium (FBS) (26400-044) were obtained from Gibco; Sequencing-grade trypsin (V5113) was purchased from Promega.

*SILAC labeling, isolation of lncRNA–protein-interacting complex*: SILAC medium (RPMI 1640 containing Leu-d3) was prepared according the manufacturer’s instruction for the following DLD-1 cell labeling. The procedure of using Leu-d3 to label the cellular proteome has been described previously^27,28^.

For RNA pull-down, equal amounts of proteins derived from Light (in normal medium) and Heavy (in Leu-d_3_ medium) cell pools were incubated with biotin-labeled anti-sense RNA and sense RNA, followed by the incubation with streptavidin beads, respectively. After pull-down, the beads from two fractions were combined and the proteins were eluted by 1 × SDS loading buffer. The eluate was then separated by 12% sodium dodecyl sulfate polyacrylamide gel electrophoresis gel and stained with Coomassie brilliant blue (CBB).

*Band excision, in-gel trypsin digestion, and peptide extraction*: The CBB-stained gel was excised as 11 bands. Following the protocol described previously^49^, Each of gel bands was cut into pieces and CBB dye was removed with 50% acetonitrile (ACN)/50 mM ammonium bicarbonate. The gel pieces were then dehydrated twice in 100% ACN for 30 min and reconstituted in the in-gel digestion buffer containing 10 ng/μl sequencing-grade trypsin (Promega, V5113) overnight at 37 °C. The tryptic peptides were extracted from the gel pieces with 50% ACN/0.1% trifluoroacetic acid (TFA) and lyophilized.

*Liquid chromatography–mass spectrometry* (*LC-MS*) *analysis*: LC-MS analysis was performed on a Nano Aquity UPLC system (Waters Corporation, MA, U.S.) connected to a quadrupole-Orbitrap mass spectrometer (Q-Exactive) (Thermo Fisher Scientific, Bremen, Germany) equipped with an online nano-electrospray ion source. Peptides were resuspended with 10 µl solvent A (5% acetonitrile, 0.1% formic acid in water). Eight microliters of peptide sample was loaded onto the Thermo Scientific Acclaim PepMap C18 column (100 μm × 2 cm, 5 μm, Thermo Fisher Scientific) with a flow of 10 μl/min for 3 min and then was separated on the analytical column (Acclaim PepMap C18, 75 μm × 15 cm, 2 μm, 100 Å) with a linear gradient. The gradient started from 2% B (90% acetonitrile, 0.1% formic acid in water) to 45% B over 75 min. The column was re-equilibrated at initial conditions for 15 min. The column flow rate was maintained at 300 nL/min and column temperature was maintained at 40 °C. The electrospray voltage of 2.2 kV versus the inlet of the mass spectrometer was used.

The Q-Exactive mass spectrometer was operated in the data-dependent mode to switch automatically between MS and MS/MS acquisition. Survey full-scan MS spectra (*m*/*z* 350–1600) were acquired with a mass resolution of 70,000, the AGC target value was set to 1 × 10^6^, and the maximum injection time was 50 ms. Fifteen most intense peaks with charge state 2–4 were selected for fragmentation using higher-energy collisional dissociation (HCD) with normalized collision energy (NCE) of 30%, the isolation window of 2 *m*/*z* was used. The HCD fragments were analyzed in the Orbitrap mass analyzer with resolution 17,500, The AGC target value was set to 2 × 10^5^, the maximum injection time was 150 ms. In all cases, one microscan was recorded using dynamic exclusion of 60 s. The spectra were recorded with Xcalibur (version 2.2 SP1) software.

*Database search, protein identification, and quantification*: The eleven of MS raw files generated by Q-Exactive were processed using MaxQuant software (version 1.5.2.8, http://www.maxquant.org/) for protein identification and quantification^50^. Data were searched using the Andromeda search engine^51^ against the Human UniProtKB/Swiss-Prot database (20,197 entries). The parameters were set as follows: (1) The minimum required peptide length was seven amino acids. (2) Trypsin cleavage specificity was applied with up to two missed cleavages allowed. (3) Variable modifications included methionine oxidation (+15.9994 Da), N-acetylation of protein N-termini (+42.0106Da), and leu-d3 (+3.0188Da). (4) Initial mass deviation of the precursor ion and fragment ions were up to 10 ppm and 0.5 Da, respectively. (5) The false discovery rate (FDR) was set to 1% at both the peptide and protein levels. (6) Minimum of peptides was set to 2. (7) Multiplicity of 2 was used, where Leu and Leu-d3 (+3.0188Da) were selected as light (L) and heavy (H) labels, respectively. (8) “Re-quantify” and “match between runs” were selected. (9) Quantification was performed using unmodified unique and razor peptides and a minimum of two counted ratios. (10) The protein ratios (*H*/*L*) were automatically calculated and normalized by MaxQuant.

To clarify the lncRNA-specific-interacting proteins from the dataset generated by MaxQuant, Perseus (version 1.5.2.6, http://www.perseusframework.org/), the bioinformatics analysis software compatible with MaxQuant, was used to determine the specific interactors by the significance *B*-value (*p* < 0.05), which is the *p-*value for detection of significant outlier protein ratios calculated on the protein subsets obtained by intensity binning^50^.

### In situ hybridization and immunohistochemical staining

The in situ detection of GLCC1 was performed on 6-μm formalinfixed, paraffin-embedded sections using DIG-labeled miRCURYTM Detection probe (Exiqon). The probe sequence of GLCC1 is listed as follows: 5′–3′ /5DigN/TACACAATTCAAAGGCAGGCAT/3Dig_N/. Briefly, the slides were hybridized with a probe (LNA-modified and DIG-labeled oligonucleotide; Exiqon) complementary to GLCC1 and after incubation with anti-DIG-AP Fab fragments conjugated to alkaline phosphatase. The hybridized probes were then detected by applying nitroblue tetrazolium/5-bromo-4-chloro-3-indolyl phosphate color substrate (Roche) to the slides. Positive controls (lncRNA AK000053, RNU6B, Exiqon) and scrambled control RNA were included for each hybridization procedure. Slides were counterstained with VECTOR® nuclear fast red counterstain (VECTOR LABOTATORIES) and analyzed with a Nikon 80i microscope and Nikon NIS-Elements F 2.3 software (Nikon).

The expression of Ki67/c-Myc/LDHA was examined with a specific antibody dilusion (Ki67: Abcam; 1:100; c-Myc: Abcam; 1:200; LDHA: Abcam; 1:600) using the LSAB+Kit (DakoCytomation).

The slides were examined independently by two investigators, who were blinded to the clinical and pathological data. Protein and lncRNA expression were quantified using a visual grading system based on the extent of staining (percentage of positive tumor cells) and the intensity of staining. For further analysis, the product (the corresponding score) of the extent and intensity grades was used to define the cutoff value for different expression levels of the proteins and lncRNA. In the correlative and survival studies, the expression levels of relevant proteins and lncRNA were classified into two categories.

### In vitro transcription and translation

LncRNA GLCC1 and *c-Myc* were cloned into pBluescript KSII downstream of the T7 promoter. The recombinant plasmids were transcripted (Promega, USA), and purified (Qigen, Germany) in vitro. The purified GLCC1, *c-Myc* RNA and other control RNAs were translated in vitro using Biotinylated leucine tRNA (Promega, USA). Biotinylated proteins were detected using a BrightStar BioDetect Kit (Ambion, USA). *c-Myc* messenger RNA (mRNA) was used as a positive translation control, and GClnc1 and water serve as negative controls.

### GST pull-down assay

The codon-optimized genes of *c-Myc* and *HSP90* (*HSP90AA1*) were synthesized by PCR-based accurate synthesis and cloned in frame with GST tag in pGEX-4T-1 vector (*Eco*RI/*Xho*I, GE Healthcare) and with His tag into pCzn1 vector (NdeI/XbaI, Zoonbio Biotechnology, China), respectively. Constructs were verified by sequencing.

GST- and His-tagged constructs were transformed into Arctic-Express competent cells, induced (IPTG,0.5 mM), and purified using either glutathione beads (GE Healthcare) or Ni2+ agarose beads (QIAGEN) according to the manufacturer’s instruction.

For the pull-down assay, 2 μg of GST or GST-tagged c-Myc proteins were mixed with 100 μl of glutathione-Sepharose 4B beads (GE Healthcare) for 1 h at 4 °C in binding buffer (20 mM Tris(pH7.5), 150 mM NaCl, 1% Triton X-100, 2 mM DTT, 1 mM EDTA). Then 1 μg of His-tagged HSP90 was added in the absence or presence of lncRNA and incubated for another 1 h at 4 °C. The beads were collected and washed with binding buffer, then 2 × SDS loading buffer was added, boiled, and subjected to the following western blot analysis. Primary antibodies used included anti-GST (CST, 1:1000) and anti-His (Abcam, 1:5000).

### Luciferase assay

Cell lines were transfected with the plasmids expressing the designated combinations of pGL3-LDHAPWT and other relevant siRNAs at 1.0 μg and 100 ng of phRL (Renilla luciferase) with Lipofectamine 2000 (Invitrogen) or DharmaFECT 1 siRNA transfection reagents (Dharmacon). Twenty-four hours after transfection, the cells were collected to detect luciferase activity using the Dual-Luciferase reporter assay system (Promega). Luciferase activity was measured by using a FLUOstar Omega (BMG LABTECH). Transfection efficiency was normalized by dividing the luciferase activity of the construct by the corresponding Renilla luciferase activity.

### Western blot

Western blot analysis was performed using standard technique^52^. An anti-β-actin antibody was used as a control for whole-cell lysates. Antibodies were purchased mainly from three manufacturers: Cell Signaling Technology, Inc. (c-Myc, LDHA, HIF-1a, P53, HSP90AA1, HSP90AB1), Abcam (Ki67, USP22, USP28). The dilution of primary antibodies was 1:1000. The uncropped and unprocessed scans of blots were shown in the Supplementary Figs. 7–11.

### Northern blot

Northern blotting was performed with 200 to 500 ng of purified poly (A) mRNA. RNAs were resolved using denaturing agarose gel electrophoresis (Ambion) and transferred to Hybond-XL membranes (GE Healthcare). GLCC1 were detected using 32P-labeled DNA probes.

### Co-immunoprecipitation

Co-immunoprecipitation was performed as described previously^53^. Briefly, both input and IP samples were analyzed by western blot using various antibodies at the indicated dilutions: HSP90 antibody (1: 1000; Cell Signal Technology and 1:500; Santa Cruz), c-Myc antibody (1:1000; Cell Signal Technology and and 1:500; Santa Cruz), and normal rabbit IgG.

### RNA immunoprecipitation

RNA immunoprecipitation (RIP) experiments were performed using the Megna RIP RNA-binding Protein Immunoprecipitation Kit (Millipore). The HSP90 antibody used for RIP was purchased from Santa Cruz. The co-precipitated RNAs were detected by reverse transcription PCR. To demonstrate that the detected RNA signals specifically bind to HSP90, total RNA (input controls) and normal rabbit IgG controls were simultaneously assayed.

### Adenovirus and plasmids construction

The control shRNA, GLCC1 shRNA, HSP90 shRNA, c-Myc shRNA, GLCC1-overexpressing adenovirus, and all plasmids were constructed by Shanghai Obio Technology Company.

### In vivo experiments

In order to clarify the effect of GLCC1 in vivo, 4-week-old male BALB/c nude mice obtained from Experimental Animal Center of Shanghai laboratory animal center were used in our study. DLD-1 cells (1.0 × 10^7^ or 5 × 10^6^ cells for establishing GLCC1-overexpressing with c-Myc or HSP90 inhibition colorectal cancer xenograft model) were injected subcutaneously into the right flank of these mice to establish the colorectal cancer xenograft model. Ten days after subcutaneous inoculation, mice were randomly divided into different groups and were injected with PBS, control shRNA or GLCC1 shRNA by ways of multipoint intratumoral injection every other day for 14 days. Tumor volume (mm^3^) was estimated by the formula: tumor volume (mm^3^) =  shorter diameter^2^ × longer diameter/^2^. All experimental procedures were approved by the Institutional Animal Care and Use Committee of Renji Hospital, School of Medicine, Shanghai Jiao Tong University.

### ChIP and high-throughput sequencing

For ChIP-Seq, chromatin was further sonicated to reduce size, and immunoprecipitated with c-Myc antibody. Library generation was performed using pooled ChIP DNA samples from three independent ChIP preparations using the Illumina protocol. Briefly, ChIP DNA fragment ends were repaired and phosphorylated using Klenow, T4 DNA polymerase and T4 polynucleotide kinase (Illumina kit components, USA). After ligation of Illumina adapters, DNA was size selected by gel purification and then PCR amplified using Illumina primers. Sequencing was performed at Genenergy Inc, Shanghai on an Illumina Hi-Seq 3000 machine. The FASTQ files were aligned to hg19 using Bowtie. Enriched regions were determined by the MACS program (http://liulab.dfci.harvard.edu/MACS/)^54^ with a default setting.

For RNA sequencing, GLCC1 siRNA were transfected into DLD-1 cells to verify the RNAi efficiency. Real-time PCR showed the expression of the GLCC1 was significantly decreased after GLCC1 siRNA transfection, compared with the control siRNA (Supplementary Fig. S2d, e). The data demonstrated that GLCC1 siRNA specifically and effectively downregulated GLCC1 expression. Therefore, we performed High-Throughput RNA sequencing after knockdown of GLCC1 in DLD-1 cells. For RNA sequencing of siRNA-infected DLD-1 cells, each sample was cleaned up on an RNeasy Mini Column (Qiagen, Limburg, Netherlands), treated with DNase, and analyzed for quality on an Agilent 2100 Bioanalyzer. Samples were on an Illumina HiSeq 3000 for 2 × 150-bp paired-end sequencing. The RNA-seq data analysis was performed according to the TopHat-HTSeq-DeSeq2 frame^55^. Briefly, reads were mapped to the human genome (hg19) using TopHat v2.0.11^56^ (http://tophat.cbcb.umd.edu) with the default options with a TopHat transcript index built from Ensembl_GRCh37. Count files of the aligned sequencing reads were generated by the htseq-count script from the Python package HTSeq with union mode, using the GTF annotation file^57^. The read counts from each sequenced sample were combined into a count file, which was subsequently used for the differential expression analysis. Differential analyses were performed to the count files using DESeq2 packages, following standard normalization procedures^58^. Genes with <5 total counts in both conditions were removed from further analysis. The RNA sequence data have been deposited in NCBIs Gene Expression Omnibus (GEO) and are accessible through GEO Series accession number GSE119866.

### Statistical analysis

All statistical analyses were performed using R-3.0.2 (http://cran.r-project.org/bin/windows/base/old/3.0.2/). Date were examined whether they were normally distributed with the One-Sample Kolmogorov–Smirnov test. If the data were normally distributed and the variation between groups were comparable, the comparisons of measurement data between the two groups were performed using Student’ *t*-test. The comparisons among three or more groups were firstly performed by one-way analysis of variance (ANOVA) test if the variation between groups were comparable. If the results showed significant difference, the Student Newman Keuls analysis was used to test the difference between the two groups. For the clinicopathologic analysis, the Chi-square test or Fisher exact test (two-sided) were performed. The Kaplan–Meier method was used to estimate overall survival. The log-rank test was used to evaluate the differences between survival curves. All *p*-values were two-sided unless otherwise specified.

### Reporting summary

Further information on research design is available in the Nature Research Reporting Summary linked to this article.

## Supplementary information

Supplementary Information

Description of Additional Supplementary Files

Supplementary Data 1

Supplementary Data 2

Supplementary Data 3

Supplementary Data 4

Supplementary Data 5

Supplementary Data 6

Supplementary Data 7

Reporting Summary

## Data Availability

The high-throughput data are available in raw data accessible via GEO number: GSE119866, GSE132887, and GSE31737. The authors declare that all the other data supporting the findings of this study are available within the article and its Supplementary Information files and from the corresponding author on reasonable request.

## References

[1] Global Burden of Disease Cancer C. (2017). Global, regional, and national cancer incidence, mortality, years of life lost, years lived with disability, and disability-adjusted life-years for 32 cancer groups, 1990 to 2015: a systematic analysis for the global burden of disease study. JAMA Oncol..

[2] Bhandari A, Woodhouse M, Gupta S (2017). Colorectal cancer is a leading cause of cancer incidence and mortality among adults younger than 50 years in the USA: a SEER-based analysis with comparison to other young-onset cancers. J. Invest. Med..

[3] Seymour MT (2011). Chemotherapy options in elderly and frail patients with metastatic colorectal cancer (MRC FOCUS2): an open-label, randomised factorial trial. Lancet.

[4] Carethers JM, Jung BH (2015). Genetics and genetic biomarkers in sporadic colorectal cancer. Gastroenterology.

[5] Siena S, Sartore-Bianchi A, Di Nicolantonio F, Balfour J, Bardelli A (2009). Biomarkers predicting clinical outcome of epidermal growth factor receptor-targeted therapy in metastatic colorectal cancer. J. Natl. Cancer Inst..

[6] Di Nicolantonio F (2008). Wild-type BRAF is required for response to panitumumab or cetuximab in metastatic colorectal cancer. J. Clin. Oncol..

[7] Hanahan D, Weinberg RA (2011). Hallmarks of cancer: the next generation. Cell.

[8] Helmlinger G, Sckell A, Dellian M, Forbes NS, Jain RK (2002). Acid production in glycolysis-impaired tumors provides new insights into tumor metabolism. Clin. Cancer Res..

[9] Kato Y (2013). Acidic extracellular microenvironment and cancer. Cancer Cell Int..

[10] Gatenby RA, Gillies RJ (2004). Why do cancers have high aerobic glycolysis?. Nat. Rev. Cancer.

[11] Satoh K (2017). Global metabolic reprogramming of colorectal cancer occurs at adenoma stage and is induced by MYC. Proc. Natl Acad. Sci. USA.

[12] Ou J (2014). Loss of abhd5 promotes colorectal tumor development and progression by inducing aerobic glycolysis and epithelial-mesenchymal transition. Cell Rep..

[13] Ponting CP, Oliver PL, Reik W (2009). Evolution and functions of long noncoding RNAs. Cell.

[14] Mercer TR, Dinger ME, Mattick JS (2009). Long non-coding RNAs: insights into functions. Nat. Rev. Genet..

[15] Kogo R (2011). Long noncoding RNA HOTAIR regulates polycomb-dependent chromatin modification and is associated with poor prognosis in colorectal cancers. Cancer Res..

[16] Zhang X (2010). Maternally expressed gene 3, an imprinted noncoding RNA gene, is associated with meningioma pathogenesis and progression. Cancer Res..

[17] Lai MC (2012). Long non-coding RNA MALAT-1 overexpression predicts tumor recurrence of hepatocellular carcinoma after liver transplantation. Med Oncol..

[18] Yang F (2012). Up-regulated long non-coding RNA H19 contributes to proliferation of gastric cancer cells. FEBS J..

[19] Sun TT (2016). LncRNA GClnc1 promotes gastric carcinogenesis and may act as a modular scaffold of WDR5 and KAT2A complexes to specify the histone modification pattern. Cancer Discov..

[20] Yang F, Zhang H, Mei Y, Wu M (2014). Reciprocal regulation of HIF-1alpha and lincRNA-p21 modulates the Warburg effect. Mol. Cell.

[21] Zheng X (2017). LncRNA wires up Hippo and Hedgehog signaling to reprogramme glucose metabolism. EMBO J..

[22] Xiang JF (2014). Human colorectal cancer-specific CCAT1-L lncRNA regulates long-range chromatin interactions at the MYC locus. Cell Res..

[23] Zheng HT (2014). High expression of lncRNA MALAT1 suggests a biomarker of poor prognosis in colorectal cancer. Int J. Clin. Exp. Pathol..

[24] Hirayama A (2009). Quantitative metabolome profiling of colon and stomach cancer microenvironment by capillary electrophoresis time-of-flight mass spectrometry. Cancer Res..

[25] Fu LN (2018). Role of JMJD2B in colon cancer cell survival under glucose-deprived conditions and the underlying mechanisms. Oncogene.

[26] Zhang J (2012). Measuring energy metabolism in cultured cells, including human pluripotent stem cells and differentiated cells. Nat. Protoc..

[27] Ong SE (2002). Stable isotope labeling by amino acids in cell culture, SILAC, as a simple and accurate approach to expression proteomics. Mol. Cell Proteom..

[28] Wang T, Gu S, Ronni T, Du YC, Chen X (2005). In vivo dual-tagging proteomic approach in studying signaling pathways in immune response. J. Proteome Res..

[29] Garcia-Carbonero R, Carnero A, Paz-Ares L (2013). Inhibition of HSP90 molecular chaperones: moving into the clinic. Lancet Oncol..

[30] Pearl LH, Prodromou C, Workman P (2008). The Hsp90 molecular chaperone: an open and shut case for treatment. Biochem J..

[31] Whitesell L, Lindquist SL (2005). HSP90 and the chaperoning of cancer. Nat. Rev. Cancer.

[32] Liu YV (2007). RACK1 competes with HSP90 for binding to HIF-1alpha and is required for O(2)-independent and HSP90 inhibitor-induced degradation of HIF-1alpha. Mol. Cell.

[33] Paul I, Ahmed SF, Bhowmik A, Deb S, Ghosh MK (2013). The ubiquitin ligase CHIP regulates c-Myc stability and transcriptional activity. Oncogene.

[34] Peng Y, Chen L, Li C, Lu W, Chen J (2001). Inhibition of MDM2 by hsp90 contributes to mutant p53 stabilization. J. Biol. Chem..

[35] Gordan JD, Thompson CB, Simon MC (2007). HIF and c-Myc: sibling rivals for control of cancer cell metabolism and proliferation. Cancer Cell.

[36] Banerji U (2005). Phase I pharmacokinetic and pharmacodynamic study of 17-allylamino, 17-demethoxygeldanamycin in patients with advanced malignancies. J. Clin. Oncol..

[37] Welcker M (2004). The Fbw7 tumor suppressor regulates glycogen synthase kinase 3 phosphorylation-dependent c-Myc protein degradation. Proc. Natl Acad. Sci. USA.

[38] Kim D (2017). Deubiquitinating enzyme USP22 positively regulates c-Myc stability and tumorigenic activity in mammalian and breast cancer cells. J. Cell Physiol..

[39] Sacco JJ, Coulson JM, Clague MJ, Urbe S (2010). Emerging roles of deubiquitinases in cancer-associated pathways. IUBMB Life.

[40] Popov N (2007). The ubiquitin-specific protease USP28 is required for MYC stability. Nat. Cell Biol..

[41] Doherty JR, Cleveland JL (2013). Targeting lactate metabolism for cancer therapeutics. J. Clin. Invest..

[42] Shim H (1997). c-Myc transactivation of LDH-A: implications for tumor metabolism and growth. Proc. Natl Acad. Sci. USA.

[43] Ooi CH (2009). Oncogenic pathway combinations predict clinical prognosis in gastric cancer. PLoS Genet..

[44] Deng N (2012). A comprehensive survey of genomic alterations in gastric cancer reveals systematic patterns of molecular exclusivity and co-occurrence among distinct therapeutic targets. Gut.

[45] Wang P (2014). The STAT3-binding long noncoding RNA lnc-DC controls human dendritic cell differentiation. Science.

[46] Li Z (2018). The LINC01138 drives malignancies via activating arginine methyltransferase 5 in hepatocellular carcinoma. Nat. Commun..

[47] Janssen AM (2000). Superoxide dismutases in gastric and esophageal cancer and the prognostic impact in gastric cancer. Clin. Cancer Res..

[48] Warburg O. (1956). On the Origin of Cancer Cells. Science.

[49] Shevchenko A, Tomas H, Havlis J, Olsen JV, Mann M (2006). In-gel digestion for mass spectrometric characterization of proteins and proteomes. Nat. Protoc..

[50] Cox J, Mann M (2008). MaxQuant enables high peptide identification rates, individualized p.p.b.-range mass accuracies and proteome-wide protein quantification. Nat. Biotechnol..

[51] Cox J (2011). Andromeda: a peptide search engine integrated into the MaxQuant environment. J. Proteome Res..

[52] Xiong H (2012). Roles of STAT3 and ZEB1 proteins in E-cadherin down-regulation and human colorectal cancer epithelial-mesenchymal transition. J. Biol. Chem..

[53] Uyama N (2006). Hepatic stellate cells express synemin, a protein bridging intermediate filaments to focal adhesions. Gut.

[54] Zhang Y (2008). Model-based analysis of ChIP-Seq (MACS). Genome Biol..

[55] Anders S (2013). Count-based differential expression analysis of RNA sequencing data using R and Bioconductor. Nat. Protoc..

[56] Kim D (2013). TopHat2: accurate alignment of transcriptomes in the presence of insertions, deletions and gene fusions. Genome Biol..

[57] Anders S, Pyl PT, Huber W (2015). HTSeq–a Python framework to work with high-throughput sequencing data. Bioinformatics.

[58] Love, M. I., Huber, W. & Anders, S. Moderated estimation of fold change and dispersion for RNA-seq data with DESeq2. *Genome Biol*. **15**, 550 (2014). 10.1186/s13059-014-0550-8 PMC4302049 25516281

