# Supplementary material for: LncRNA GLCC1 promotes colorectal carcinogenesis and glucose metabolism by stabilizing c-Myc
Source: Nat Commun. 2019 Aug 2;10:3499. doi: 10.1038/s41467-019-11447-8 (PMC6677832; doi:10.1038/s41467-019-11447-8)
Supplement: Supplementary file 1 — Supplementary Information [file 41467_2019_11447_MOESM1_ESM.pdf]

SUPPLEMENTARY MATERIAL

Supplementary figures

Supplementary figure 1. LncRNA candidate AF339830 is clinically relevant in colorectal cancer. 1

Supplementary figure 2. LncGLCC1 is an oncogenic lncRNA in colorectal cancer. 3

Supplementary figure 3. LncGLCC1-induced cell survival depends on glycolytic metabolism. 4

Supplementary figure 4. lncGLCC1 interacts with HSP90 and regulate the stability of c-Myc. 5

Supplementary figure 5. C-Myc participate in the biological function of lncGLCC1 in CRC cells 6

Supplementary figure 6. LncGLCC1 coordinates the localization of c-Myc genome-wide 7

Supplementary figure 7. Uncropped scans of western blots or PCR gel presented in Figure 4c-4h. 8

Supplementary figure 8. Uncropped scans of western blots presented in Figure 4j-4n. 9

Supplementary figure 9. Uncropped scans of western blots or PCR gel presented in Figure 6. 10

Supplementary figure 10. Uncropped scans of western blots or PCR gel presented in Supplementary Figure 1-4f. 11

Supplementary figure 11. Uncropped scans of western blots presented in Supplementary Figure 4g-5a. 12

Supplementary tables

Supplementary Table 1. SiRNA sequences. 13

Supplementary Table 2. All primers for real-time PCR assay.

Supplementary Fig.1

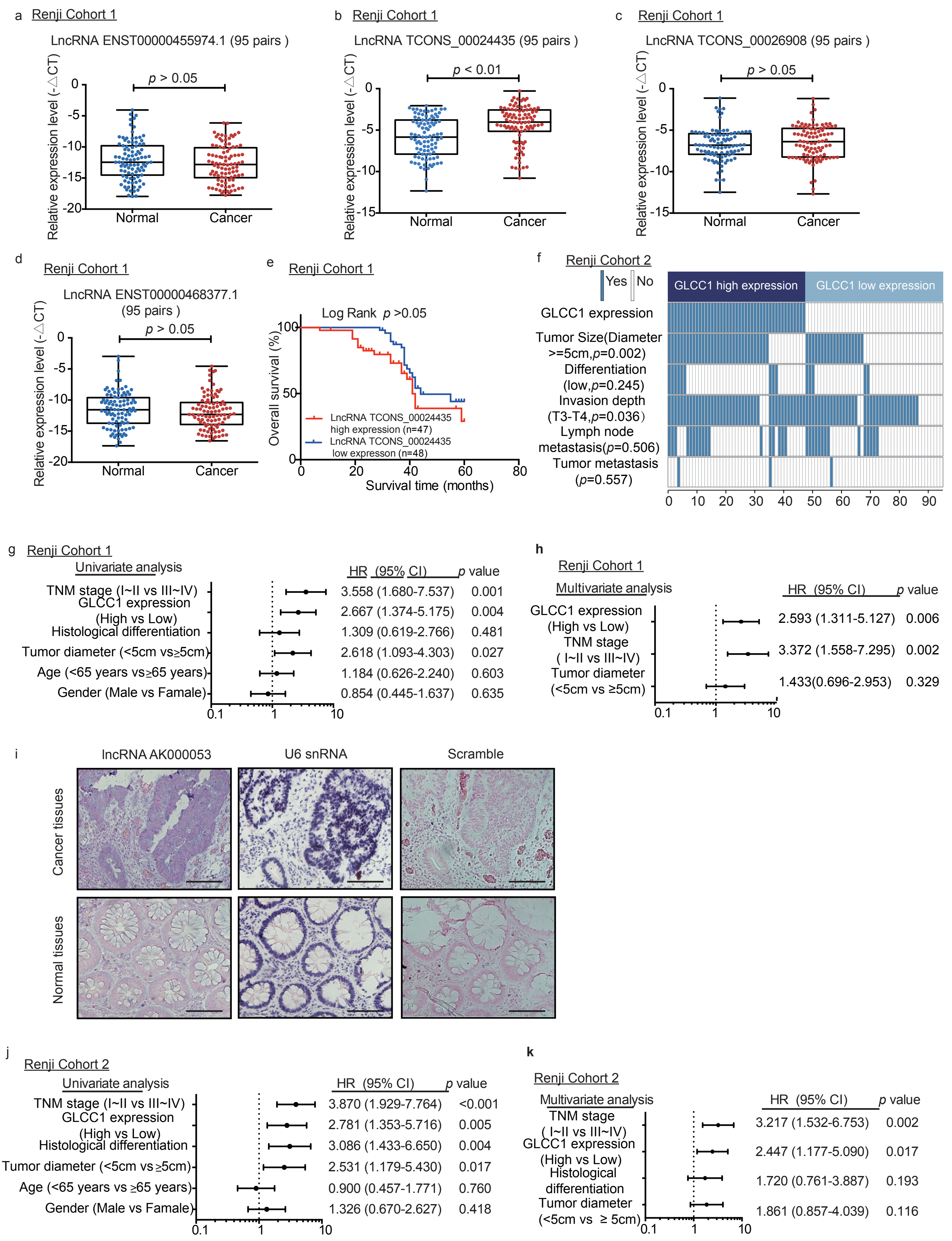

Supplementary Fig. 1

l

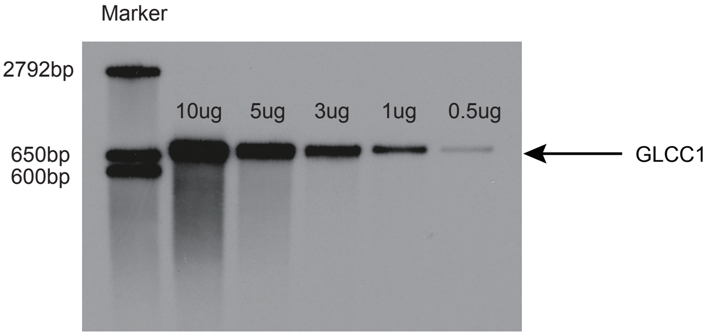

m

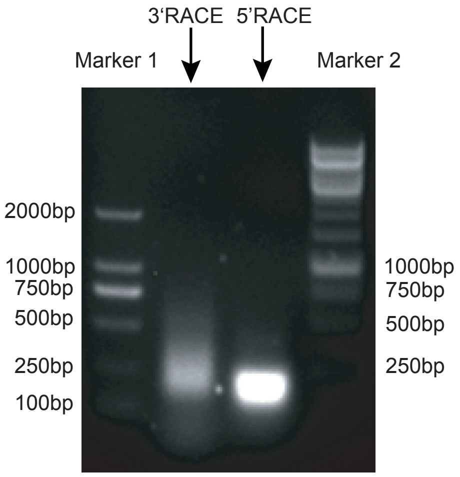

n

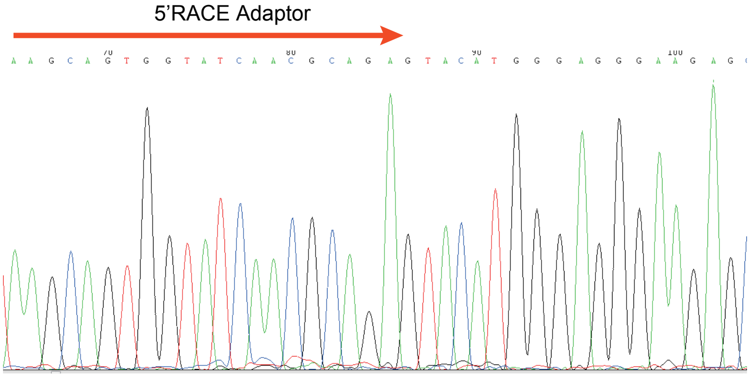

GLCC1 sequence

agggaagagcaacaccttgaaataggcactgttgtcaccagtaggtcaacaaactaaatgcctgcctttgaattgtgtacagcgcag
agaaggactgttttccagcatggttgctcttggttggtgtacagacacagtcttaccattcacactctggtgaacagtgtcctctgttgggaatagtg
atacttggatgtttctctgagccagaaaaaagaaaatgtaaggagagagagattgacttctttttaaatcttgcccaccagtgtcactgcctttcacc
cagcattcctgcattttagattaaagagcctggttttcacttgaagtttctctgccttagctgcacgttgcaattttctgagatattttaaaaattagtgat
gccgggaccctgaccctagggattttgatctcattggtcttgagtgctccttgggcttcccagctgtttgtactgggcagccagggtcacttcttacc
caggctgtctcgaatctagtggaatacagtaaaagtgttagctaaatttttggcttgcttgcagagtaagaaaaacatcacaaacaaactgcttttc
ttgatgcctgtgatttgtacttttcagccttaggtacttttttttgaaaaaaaaaaaaaaaaaa

o

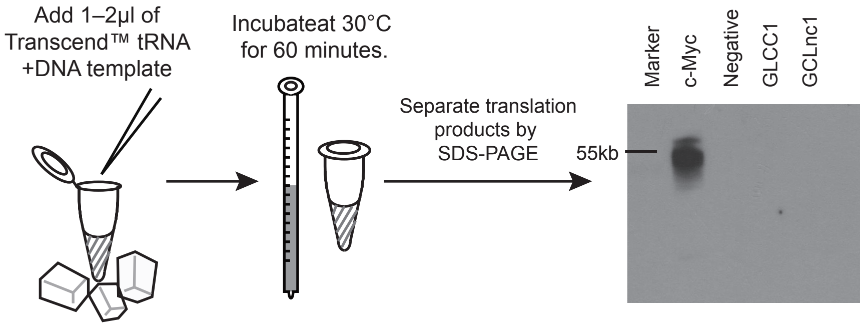

p

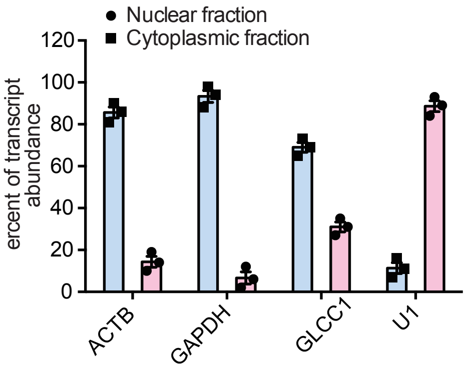

Supplementary Fig.1 LncRNA candidate AF339830 is clinically relevant in colorectal cancer
a-d. Statistical analysis of lncRNA ENST00000455974.1, TCONS_00024435, TCONS_00026908 and ENST00000468377.1 expression in 95 pairs of colorectal cancer and normal tissues (cohort 1, paired t test). e. Survival was analyzed and compared between patients with low and high levels of lncRNA TCONS_00024435 in 95 patients with colorectal cancer (cohort 1), log-rank test. f. Comparing different tumor size, differentiation and TNM stage between GLCC1 high-expression and lncGLCC1 low-expression tumors of cohort 2. The heatmap illustrates the association of different clinical characters with GLCC1 high and low-expression tumors. Statistical significance was performed by the $\chi 2$ test. g-h. Univariate analysis (g) and multivariate analysis (h) of GLCC1 were performed in cohort 1. All the bars correspond to 95% confidence intervals. i. Representative images of different RNA expression in colorectal cancer and adjacent colorectal tissues using ISH. lncRNA AK000053 serves as positive control. U6 snRNA serves as methodologically positive control and Scramble snRNA serves as negative control. The purple staining represents positive signal. The red color means nuclear staining and represents negative signal. Scale bar indicates 100μm. j-k.Univariate analysis(j) and multivariate analysis(k) of GLCC1 were performed in cohort 2. All the bars correspond to 95% confidence intervals. l. Expression of GLCC1 in DLD-1 cells was quantified by Northern blotting. m. Representative image of PCR products from the 5’-Race and 3’-Race are shown. n. Representative image of the sequence of 5’-Race PCR products of GLCC1 are shown. An arrow marks the boundary between the universal anchor primer and GLCC1. The GLCC1 sequence is shown at the bottom. o. In vitro translation assays for GLCC1. The transcript of GLCC1 was cloned and tested for protein coding capacity using an in vitro translation assay. C-Myc is used as a positive control. GClnc1 and water serve as negative controls. GLCC1 transcript does not generate a protein in this assay. p. Expression of GLCC1 in cytoplasmic and nuclear fractionations of colorectal cancer cells. U1 RNA serves as a positive control for nuclear gene expression. Data are presented as the mean ± SE.

Supplementary Fig. 2

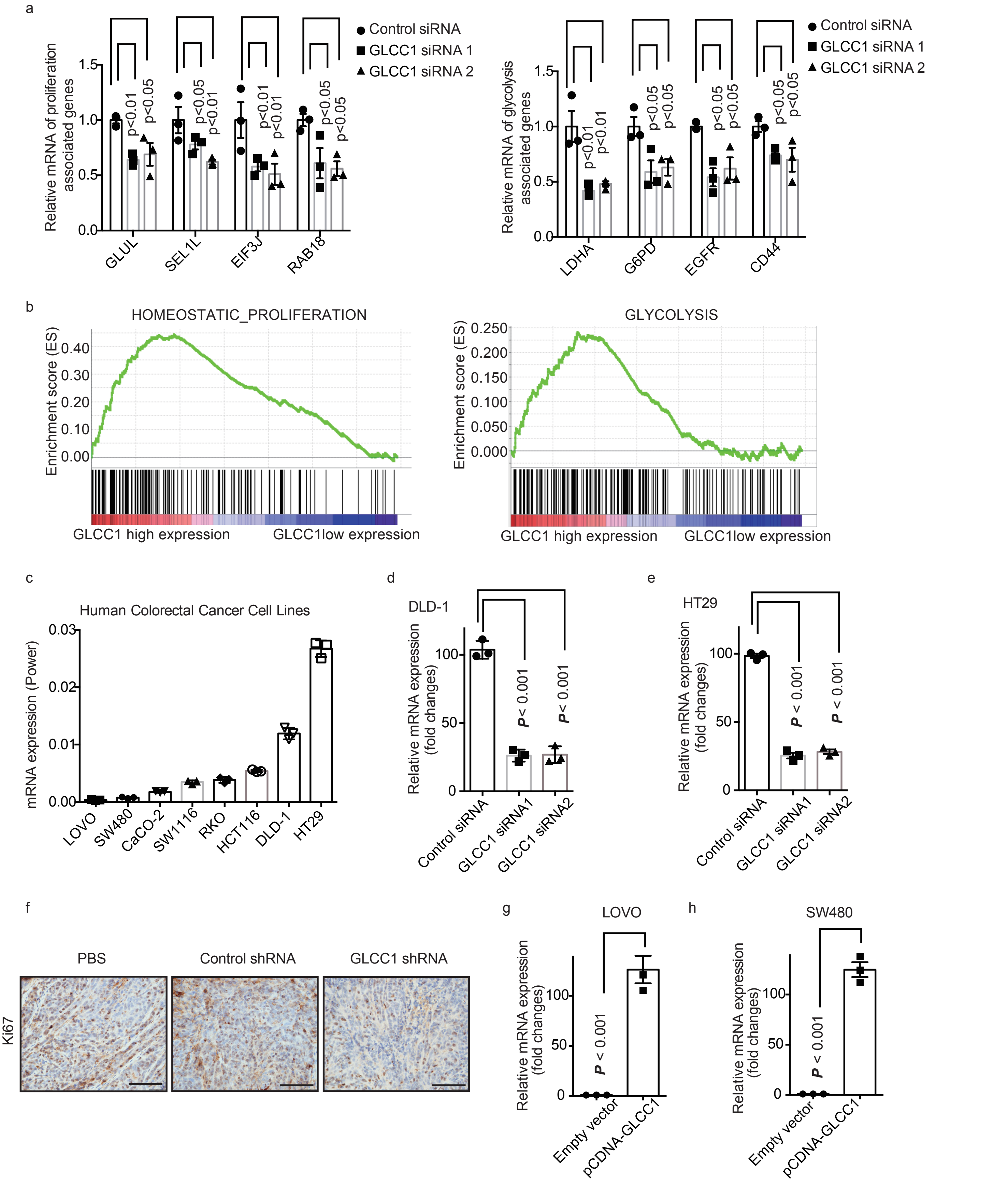

Supplementary Fig. 2 GLCC1 is an oncogenic lncRNA in colorectal cancer.
a. The mRNA levels of the tumorigenesis and glycolysis related genes were measured in colorectal cells after transfection of GLCC1 siRNAs1/2 or control siRNA. n = 3. b. Overview of GSEA used to identify the differential gene profiles between high GLCC1 expression and low GLCC1 expression colorectal tissues in GEO31737 set. c. GLCC1 expression was quantified by real-time PCR in different colorectal cells. n = 3. d-e. The downregulation efficiency of GLCC1 siRNA1/2 was confirmed in DLD-1 (d) and HT29 (e) cells. f. The representative IHC staining image of Ki67 in the three groups of xenografts is shown. Scale bar indicates 100μm. g-h. The upregulation efficiency of lncGLCC1 overexpression plasmid was confirmed in LoVo (g) and HT29 (h) cells. Data in c-e, g-h are presented as the mean ± SE. P-values were calculated by One-way ANOVA followed by SNK multiple comparison test.

Supplementary Fig. 3

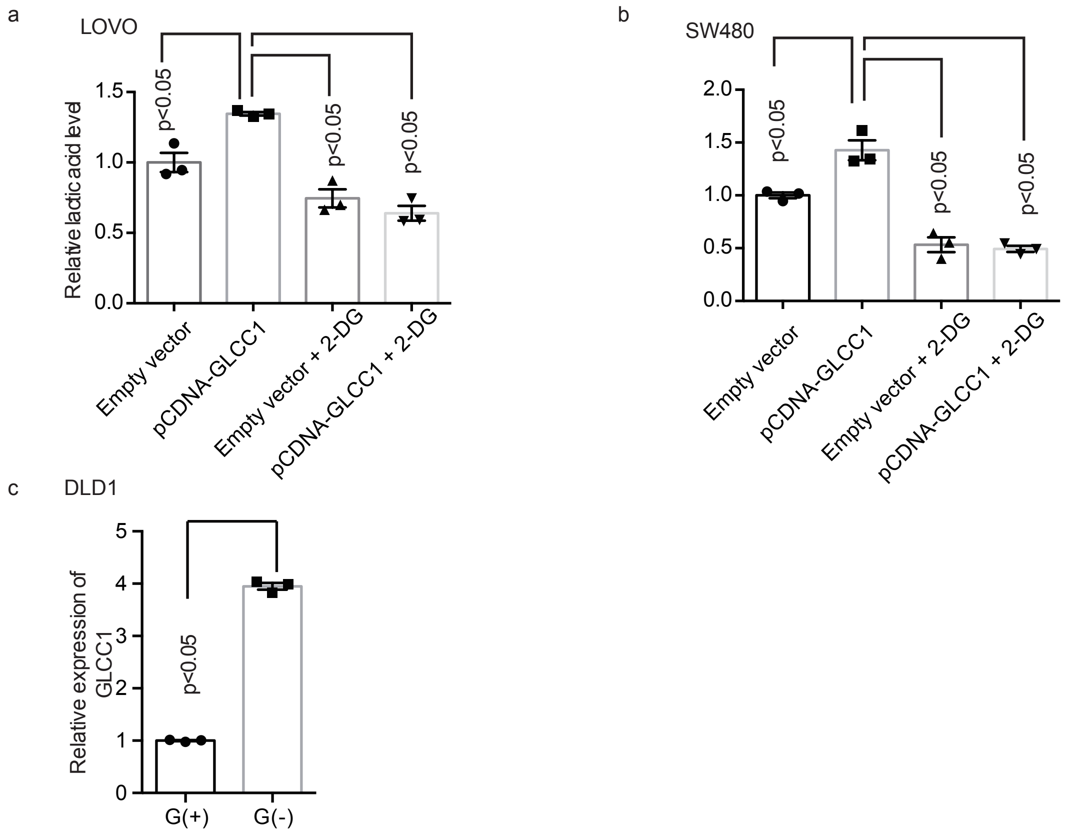

Supplementary Fig,3 GLCC1-induced cell survival depends on glycolytic metabolism

a-b. The relative lactic acid level was detected in LoVo (a) and SW480 (b) cells upon transfected with control plasmid, GLCC 1 overexpression plasmid, 2-DG, 2-DG and GLCC1 overexpression plasmid (n = 3). 2-DG, 2-deoxyglucose. c. Real time PCR was performed to detect GLCC1 expression with or without glucose treatment. Data are presented as the mean ± SE.P-values were calculated by One-way ANOVA followed by SNK multiple comparison test.

Supplementary Fig. 4

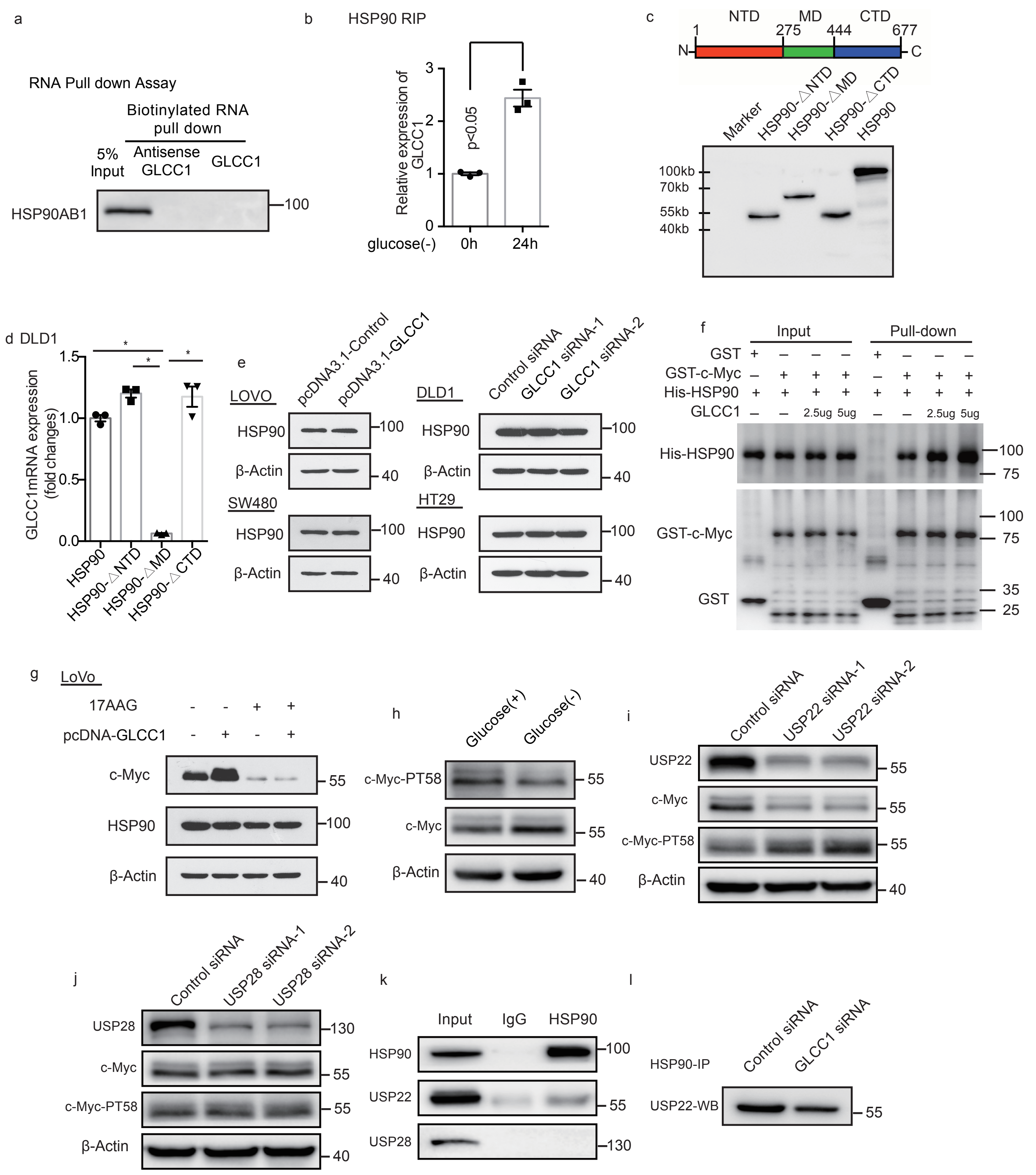

Supplementary Fig. 4 lncGLCC1 interacts with HSP90 and regulate the stability of c-Myc

a. Western blot of HSP90AB1 from lncGLCC1 pull-down assays are shown. b. Real time PCR was performed to detect GLCC1 expression after RIP assay in response to glucose starving or no treatment. log-rank test.c. Western blot assay was performed to detect different truncated-HSP90 expression in DLD-1 cells. d. Real time PCR assay was performed to detect the binding efficiency between different mutated HSP90 protein with lncRNA GLCC1 in RIP assay. P-values were calculated by One-way ANOVA followed by SNK multiple comparison test. *p<0.05.e. Western blot of HSP90 from control and lncGLCC1 overexpression plasmid in LoVo and SW480 cells (left panel). Western blot of HSP90 from control and lncGLCC1 siRNA1/2 transfection in HT29 and DLD-1 cells (right panel). f. GST pull-down assay was performed to measure the interaction between c-MYC with HSP90 (HSP90AA1). g. Western blot of c-Myc and HSP90 expression in different treatment group in LoVo cells. h. Western blot assay was used to detect pT58-c-Myc and c-Myc expression after different treatment. i. Western blot assays were performed to detect USP22, MYC and MYC-PT58 expressions with USP22 siRNA transfection in DLD1 CRC cells. j. Western blot assays were performed to detect USP28, MYC and MYC-PT58 expressions with USP28 siRNA transfection in DLD1 CRC cells. k. Co-immunoprecipitation detected the interaction of HSP90 and USP22 in the DLD-1 cells. l. Co-immunoprecipitation detected the interaction of HSP90 and USP22 in the DLD-1 cells transfected with control siRNA or GLCC1 siRNA. Data in b and d are presented as the mean ± SE.

Supplementary Fig. 5

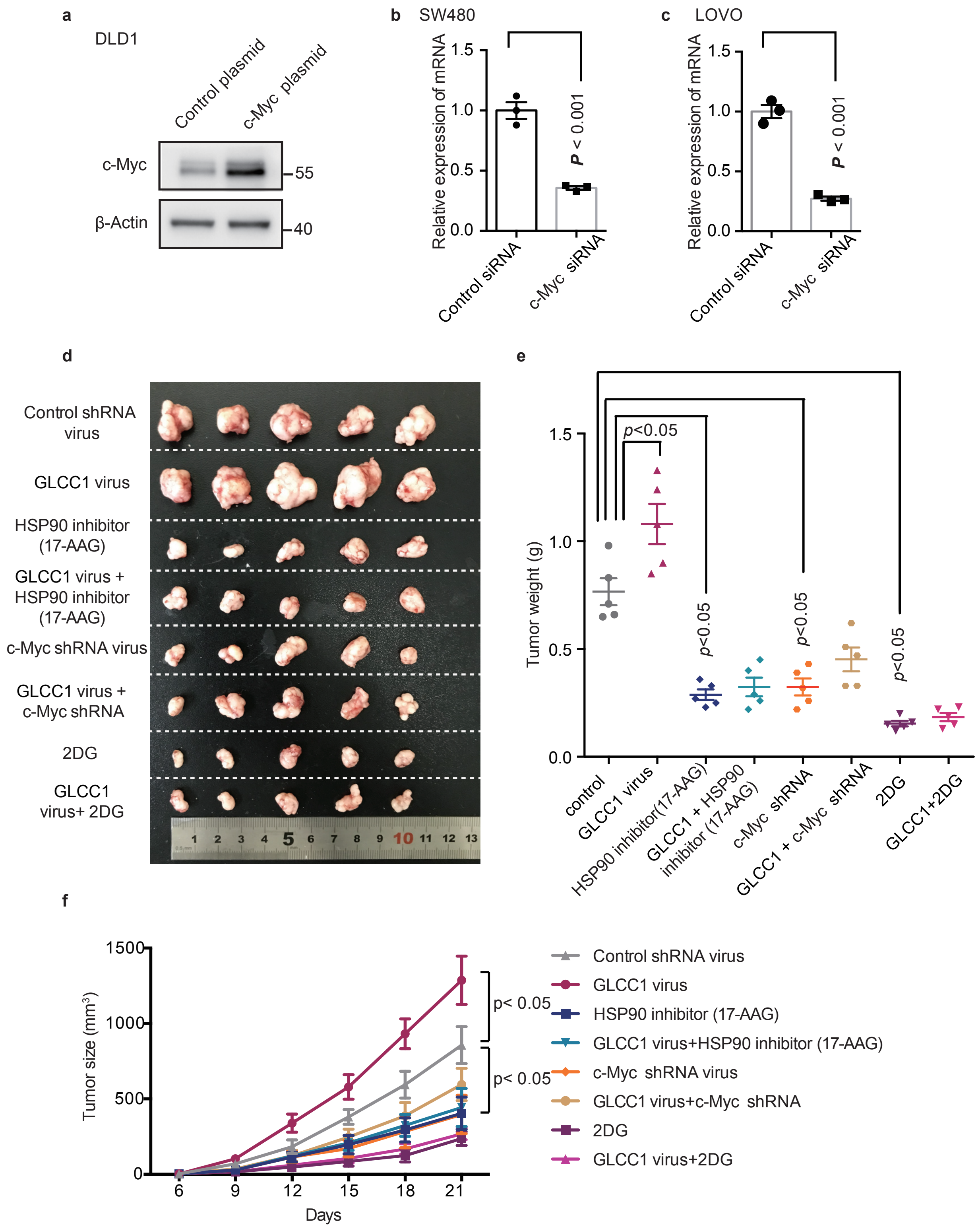

Supplementary Fig. 5 C-Myc participate in the biological function of GLCC1 in CRC cells
a. The overexpression efficiency of MYC plasmid in DLD1 cells was confirmed by western blot. b-c. The downregulation efficiency of MYC siRNA in SW480 (b) and LoVo (c) cells was confirmed by real-time PCR. d. Representative data of tumors in xenograft model was shown after injection of control shRNA adenovirus, GLCC1 overexpressing adenovirus, HSP90 inhibitor (17AAG), GLCC1 overexpressing adenovirus and inhibitor (17AAG), MYC shRNA adenovirus, GLCC1 overexpressing adenovirus and MYC shRNA adenovirus, 2-DG, GLCC1 overexpressing adenovirus and 2-DG, n = 5. e. Tumor volume was measured in the xenograft mouse model after different treatments. n =5. f. Tumor weight was measured in mice after different treatments. n = 5. Data are presented as the mean ± SE. P-values were calculated by One-way ANOVA followed by SNK multiple comparison test.

Supplementary Fig. 6

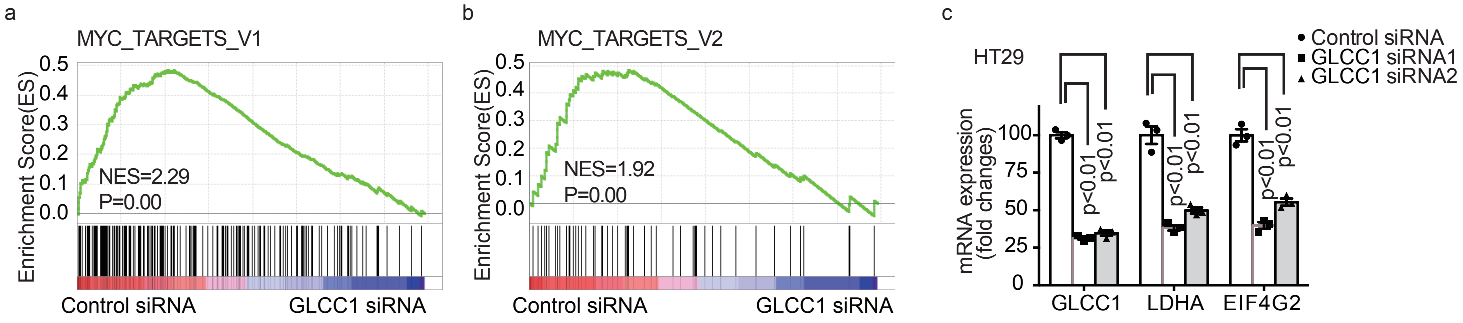

Supplementary Fig.6 GLCC1 coordinates the localization of c-Myc genome-wide

a-b. Overview of GSEA used to identify the differential gene profiles between GLCC1 siRNA transfection colorectal cancer cells and controls.

c. Real-time PCR of target genes were performed in HT-29 cells after transfection of GLCC1 siRNA1/2; n = 3. Data are presented as the mean ± SE. P-values were calculated by One-way ANOVA followed by SNK multiple comparison test.

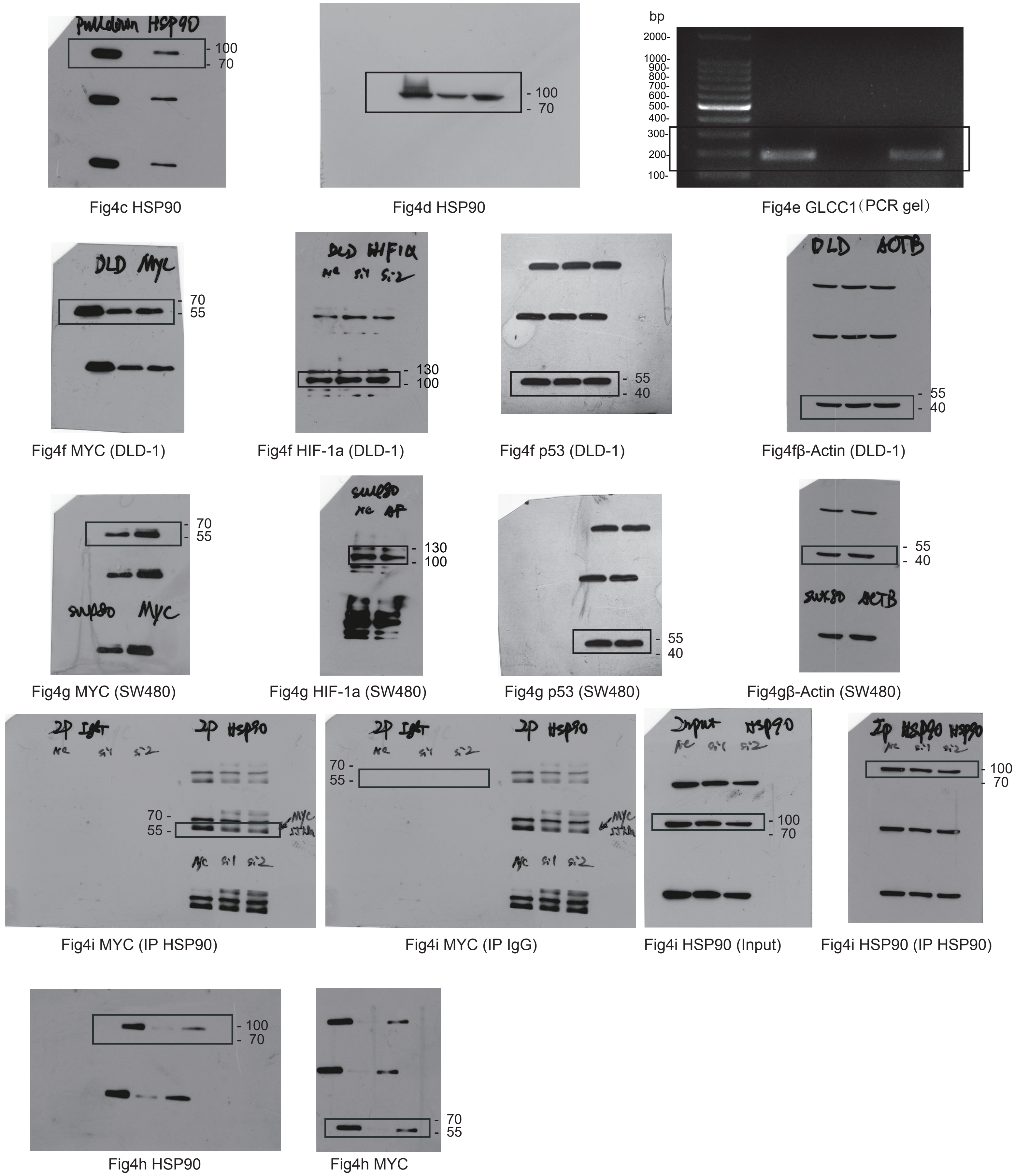

Supplementary Figure 7. Uncropped scans of western blots or PCR gel presented in Figure 4c-4h.

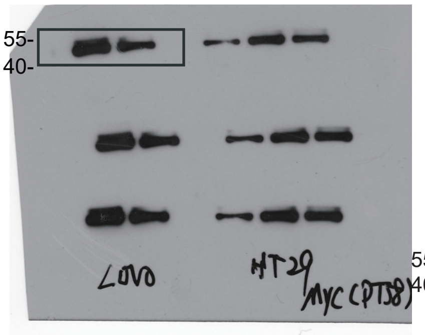

Fig4j pMYC (LoVo)

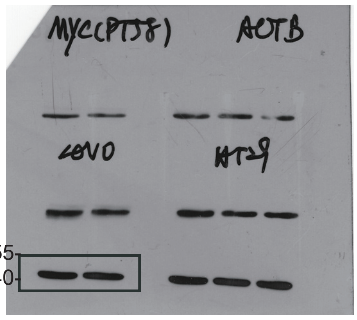

Fig4jβ-Actin (LoVo)

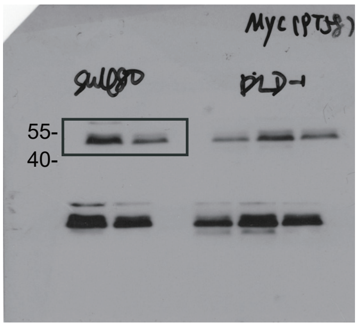

Fig4j pMYC (SW480)

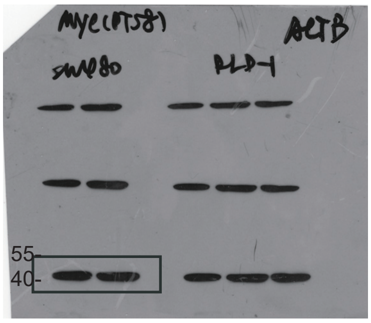

Fig4jβ-Actin (SW480)

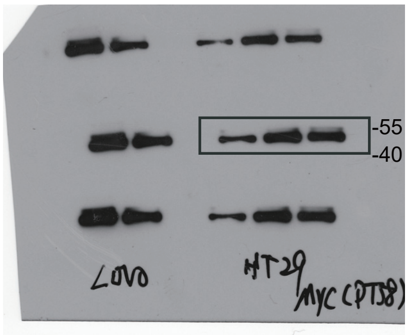

Fig4k pMYC (HT29)

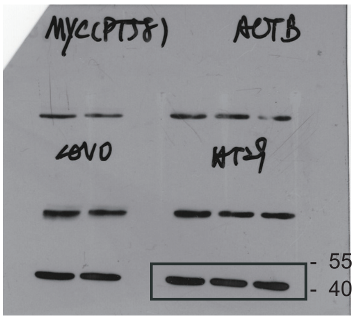

Fig4kβ-Actin (HT29)

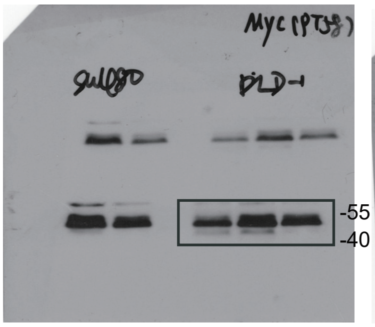

Fig4k pMYC (DLD-1)

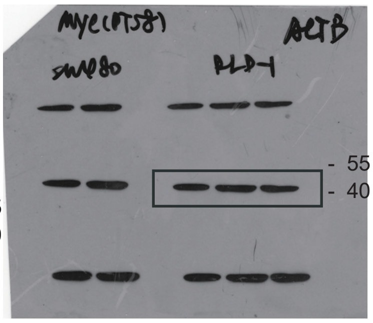

Fig4kβ-Actin (DLD-1)

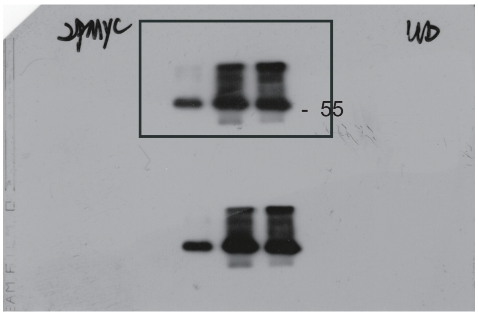

Fig4l Ub

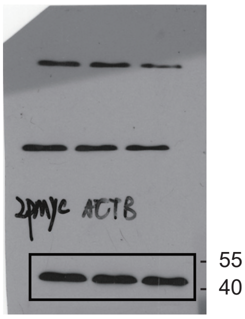

Fig4l β-Actin

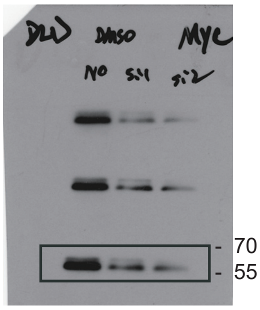

Fig4m MYC (DLD-1) Left

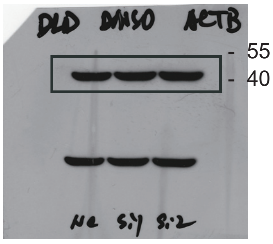

Fig4mβ-Actin (DLD-1) Left

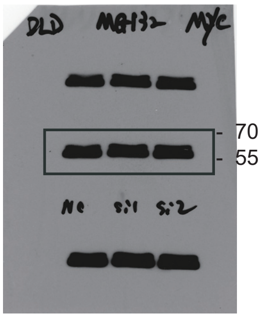

Fig4m MYC (DLD-1) Right

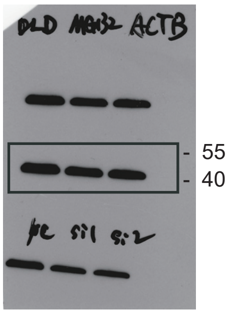

Fig4mβ-Actin(DLD-1) Right

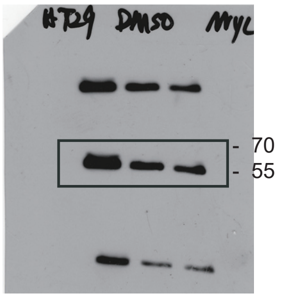

Fig4n MYC (HT29) Left

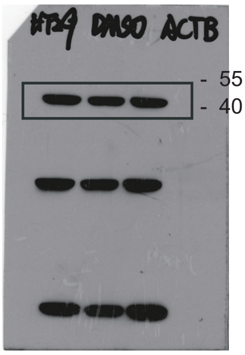

Fig4nβ-Actin (HT29) Left

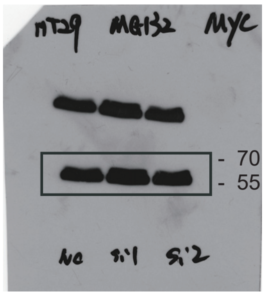

Fig4n MYC (HT29) Right

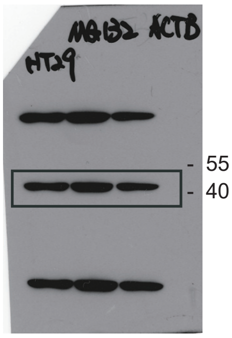

Fig4nβ-Actin(HT29) Right

Supplementary Figure 8. Uncropped scans of western blots presented in Figure 4j-4n.

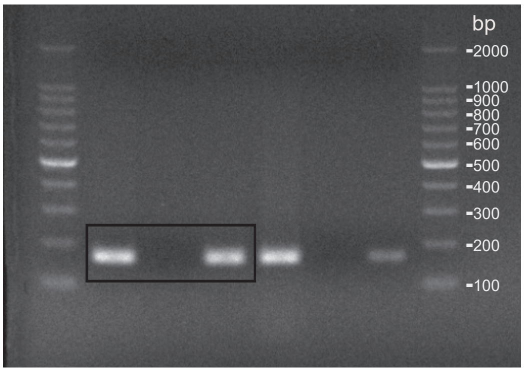

Fig6e LDHA（PCR gel）

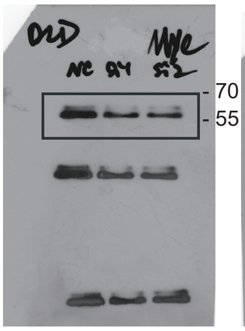

Fig6i MYC

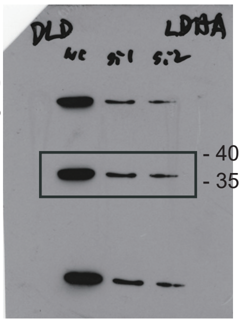

Fig6i LDHA

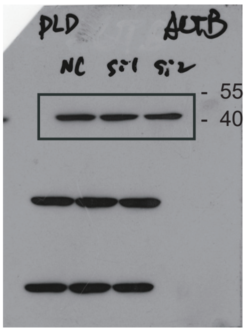

Fig6iβ-Actin

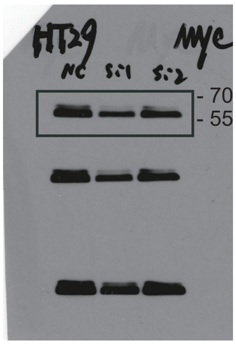

Fig6j MYC

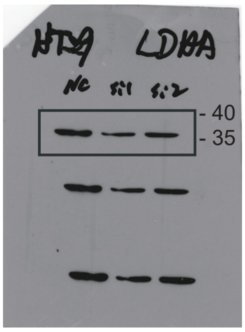

Fig6j LDHA

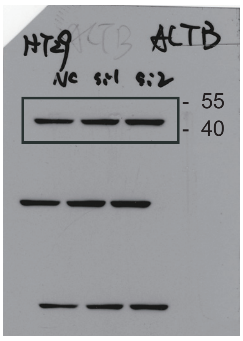

Fig6jβ-Actin

Supplementary Figure 9. Uncropped scans of western blots or PCR gel presented in Figure 6.

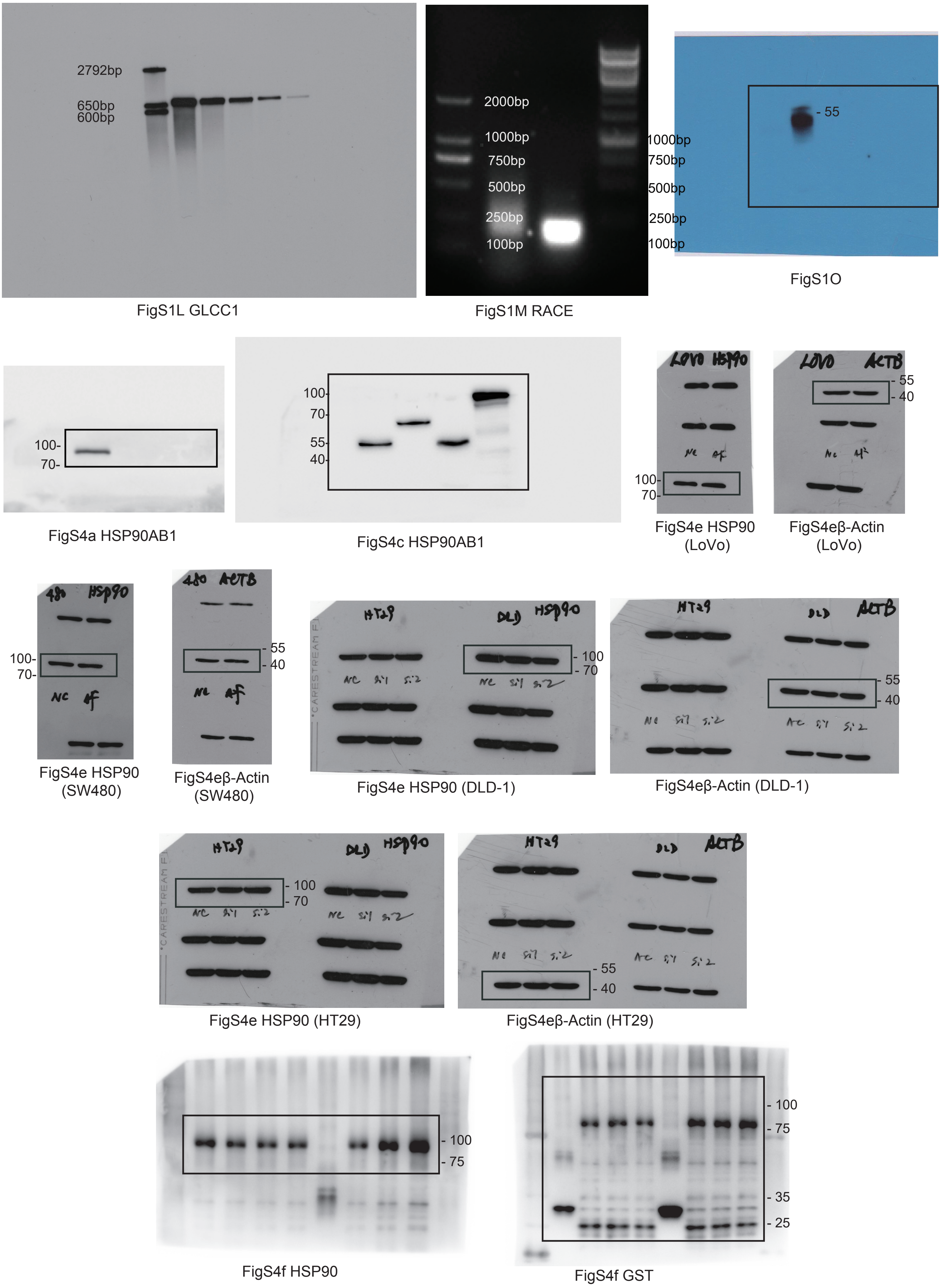

Supplementary Figure 10. Uncropped scans of western blots or PCR gel presented in Supplementary Figure 1-4f.

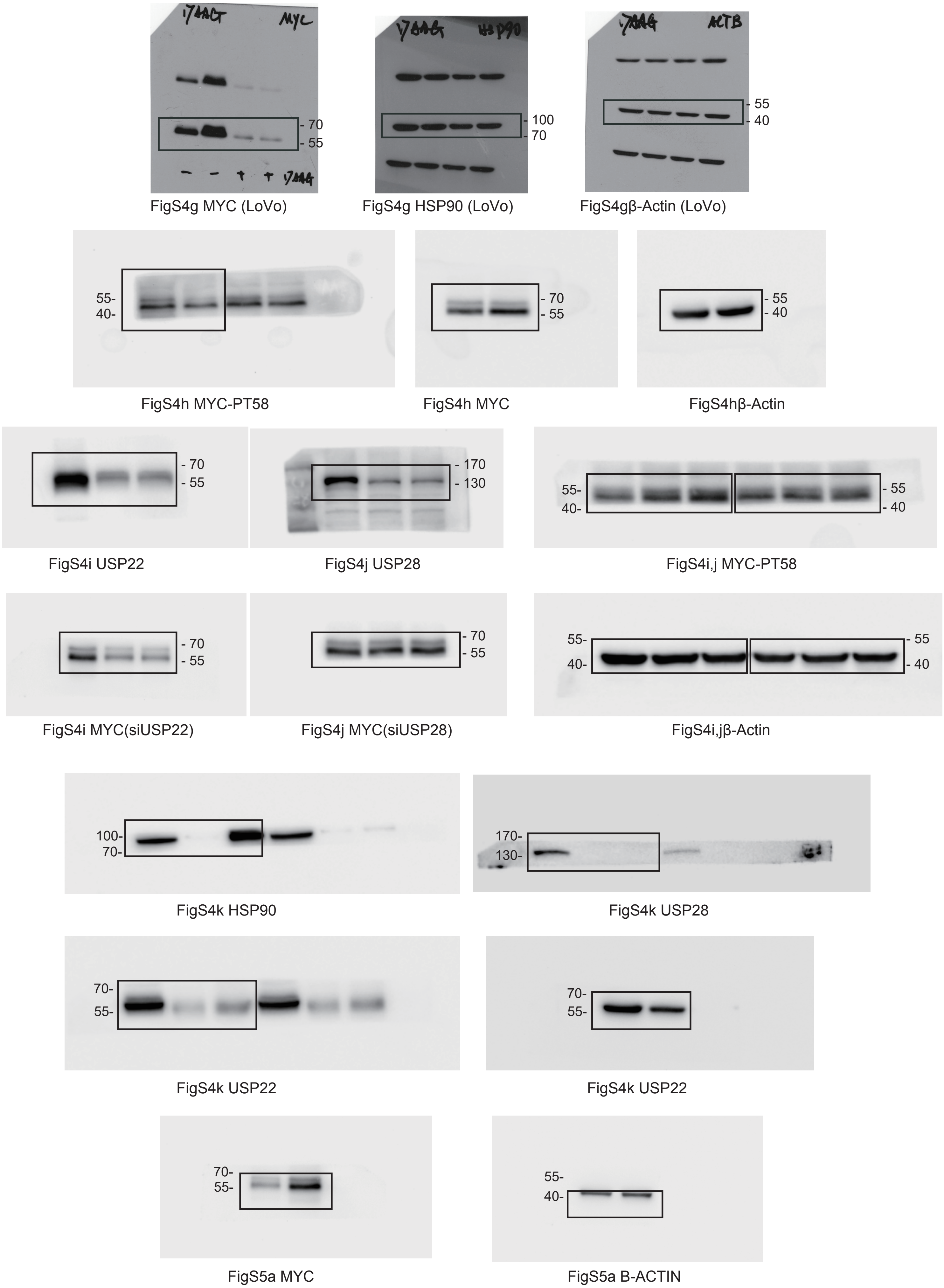

Supplementary Figure 11. Uncropped scans of western blots presented in Supplementary Figure 4g-5a.

Supplementary Table 1. SiRNA sequences.

Gene	sense（5'-3'）	antisense（5'-3'）
GLCC1 siRNA1	GGAAGAGCAACACCUUGAATT	UUCAAGGUGUUGCUCUUCCTT
GLCC1 siRNA2	CCAGGCUGUCUCGAAUCUATT	UAGAUUCGAGACAGCCUGGTT
HSP90AA1 siRNA1	GCUGGUGCAGAUAUCUCUATT	UAGAGAUAUCUGCACCAGCTT
HSP90AA1 siRNA2	GGAGAAGGAACGUGAUAAATT	UUUAUCACGUUCCUUCUCCTT
MYC siRNA1	GUGCAGCCGUAUUUCUACUTT	AGUAGAAAUACGGCUGCACTT
MYC siRNA2	GAACACACAACGUCUUGGATT	UCCAAGACGUUGUGUGUUCTT
LDHA siRNA1	CGGUUGCAAUCUGGAUUCATT	UGAAUCCAGAUUGCAACCGTT
LDHA siRNA2	CCCAGAUUUAGGGACUGAUTT	AUCAGUCCCUAAAUCUGGGTT
USP28 siRNA1	GCAAGGAGCUUAUUCGAAATT	UUUCGAAUAAGCUCCUUGCTT
USP28 siRNA2	GGAGGCCUGAGAAAUGUUATT	UAACAUUUCUCAGGCCUCCTT
USP22 siRNA1	GGAGAAAGAUCACCUCGAATT	UUCGAGGUGAUCUUUCUCCTT
USP22 siRNA2	GGAGAAAGAUCACCUCGAATT	UUCGAGGUGAUCUUUCUCCTT

Supplementary Table 2. All primers for real-time PCR assay

Gene	Former	Reverse
ACTB	CCTTGCACATGCCGGAG	GCACAGAGCCTCGCCTT
GLCC1	CAGAGAAGGACTGTTTTCCA	CATCCAAGTATCACTATTCCCA
LDHA	ATGGCAACTCTAAAGGATCAGC	CCAACCCCAACAACTGTAATCT
c-Myc	GTCAAGAGGCGAACACACAAC	TTGGACGGACAGGATGTATGC
U1	GAAACTCGACTGCATAATTTGTGGTAG	CTTGGCGTACAGTCTGTTTTTGAAACTC
GAPDH	GCATTGCCCTCAACGACCAC	CCACCACCCTGTTGCTGTAG
EIF4G2	GGCCTGCTCAGTCGTTCCTAATG	TTGAGTGCGTGGTGGTTGTGC
GLUL	TCGATGGTACTGGAGAAGGACTGC	CATGGCAGCAGGCACGAGATAC
SEL1L	CACAGAATATCCAGGCAGCGAGAG	AACACCAAGTCCAGAGGCATACAG
EIF3J	CGCTTTCTCCGTGGAAGACC	TCCTTGACGTCCTCGTCCTC
RAB18	TGGACGAGGACGTGCTAACCAC	TGAACCTCAAGAGCAGGCTGGAC
G6PD	AGTACGATGATGCAGCCTCCTACC	CTTCTCCACGATGATGCGGTTCC
EGFR	GTGTGCCACCTGTGCCATCC	GCCACCACCAGCAGCAAGAG
CD44	ACAAGCACAATCCAGGCAACTCC	TGGTGTTGTCCTTCCTTGCATTGG
