# Supplementary material for: LncRNA GLCC1 promotes colorectal carcinogenesis and glucose metabolism by stabilizing c-Myc
Source: Nat Commun. 2019 Aug 2;10:3499. doi: 10.1038/s41467-019-11447-8 (PMC6677832; doi:10.1038/s41467-019-11447-8)
Supplement: Supplementary file 2 — Description of Additional Supplementary Files [file 41467_2019_11447_MOESM2_ESM.pdf]

Description of Additional Supplementary Files

File name: Supplementary Data 1

Description: Identification of five lncRNAs candidates upregulation from CRC and glucose-free treated cells

File name: Supplementary Data 2

Description: Clinical information of 95 cases CRC patients (cohort 1)

File name: Supplementary Data 3

Description: Clinical information of 90 cases CRC patients (cohort 2)

File name: Supplementary Data 4

Description: RNA-seq analysis in DLD1 cells after GLCC1 shRNA and control shRNA transfection

File name: Supplementary Data 5

Description: The top 15 proteins in anti-sense RNA group bands specific to GLCC1 identified by Liquid Chromatography-Mass Spectrometry (LC-MS) analysis

File name: Supplementary Data 6

Description: Differential binding genes of c-Myc promoter occupancy in DLD-1 cells transfected with control shRNA and LncGLCC1 shRNA

File name: Supplementary Data 7

Description: The sequence of all recombination Plasmids
