# Supplementary material for: LncRNA GLCC1 promotes colorectal carcinogenesis and glucose metabolism by stabilizing c-Myc
Source: Nat Commun. 2019 Aug 2;10:3499. doi: 10.1038/s41467-019-11447-8 (PMC6677832; doi:10.1038/s41467-019-11447-8)
Supplement: Supplementary file 10 — Reporting Summary [file 41467_2019_11447_MOESM10_ESM.pdf]

nature research

Corresponding author(s): Jing-Yuan Fang

Last updated by author(s): Jun 20, 2019

Reporting Summary

Nature Research wishes to improve the reproducibility of the work that we publish. This form provides structure for consistency and transparency in reporting. For further information on Nature Research policies, see Authors & Referees and the Editorial Policy Checklist.

Statistics

For all statistical analyses, confirm that the following items are present in the figure legend, table legend, main text, or Methods section.

n/a	Confirmed	
☐	☒	The exact sample size (n) for each experimental group/condition, given as a discrete number and unit of measurement
☐	☒	A statement on whether measurements were taken from distinct samples or whether the same sample was measured repeatedly
☐	☒	The statistical test(s) used AND whether they are one- or two-sided<br>*Only common tests should be described solely by name; describe more complex techniques in the Methods section.*
☐	☒	A description of all covariates tested
☐	☒	A description of any assumptions or corrections, such as tests of normality and adjustment for multiple comparisons
☐	☒	A full description of the statistical parameters including central tendency (e.g. means) or other basic estimates (e.g. regression coefficient) AND variation (e.g. standard deviation) or associated estimates of uncertainty (e.g. confidence intervals)
☒	☐	For null hypothesis testing, the test statistic (e.g. F, t, r) with confidence intervals, effect sizes, degrees of freedom and P value noted<br>*Give P values as exact values whenever suitable.*
☒	☐	For Bayesian analysis, information on the choice of priors and Markov chain Monte Carlo settings
☒	☐	For hierarchical and complex designs, identification of the appropriate level for tests and full reporting of outcomes
☐	☒	Estimates of effect sizes (e.g. Cohen's d, Pearson's r), indicating how they were calculated

Our web collection on statistics for biologists contains articles on many of the points above.

Software and code

Policy information about availability of computer code

Data collection	Full detail of softwares used for data collection (including producer and version) can be found in the Methods section.
Data analysis	Full detail of softwares used for data analysis (including producer and version) can be found in the Methods section.

For manuscripts utilizing custom algorithms or software that are central to the research but not yet described in published literature, software must be made available to editors/reviewers. We strongly encourage code deposition in a community repository (e.g. GitHub). See the Nature Research guidelines for submitting code & software for further information.

Data

Policy information about availability of data

All manuscripts must include a data availability statement. This statement should provide the following information, where applicable:

- Accession codes, unique identifiers, or web links for publicly available datasets
- A list of figures that have associated raw data
- A description of any restrictions on data availability

The RNA-seq data are available in raw data accessible via GEO number: GSE119866. The authors declare that all the other data supporting the findings of this study are available within the article and its Supplementary Information files and from the corresponding author on reasonable request.

Field-specific reporting

Please select the one below that is the best fit for your research. If you are not sure, read the appropriate sections before making your selection.

☒ Life sciences ☐ Behavioural & social sciences ☐ Ecological, evolutionary & environmental sciences

October 2018

Life sciences study design

All studies must disclose on these points even when the disclosure is negative.

Sample size	Sample size was chosen to ensure an adequate statistical power.
Data exclusions	No data were excluded from analysis.
Replication	Reproducibility of experimental findings was assessed by performing experiments with independent biological replicates separately collected. Experimental variation is reported in the applicable figures as standard error of the mean.
Randomization	Not relevant
Blinding	Data underlying Figures 1f-g and 6k-i were collected with blinding.

Reporting for specific materials, systems and methods

We require information from authors about some types of materials, experimental systems and methods used in many studies. Here, indicate whether each material, system or method listed is relevant to your study. If you are not sure if a list item applies to your research, read the appropriate section before selecting a response.

Materials & experimental systems

n/a	Involved in the study	
☐	☒	Antibodies
☐	☒	Eukaryotic cell lines
☒	☐	Palaeontology
☐	☒	Animals and other organisms
☒	☐	Human research participants
☒	☐	Clinical data

Methods

n/a	Involved in the study	
☐	☒	ChIP-seq
☒	☐	Flow cytometry
☒	☐	MRI-based neuroimaging

Antibodies

Antibodies used	All antibodies are commercial. The reference and dilution is described in Method section.
Validation	Antibody validations were performed as described on the manufacturers' websites and were supported by multiple publications.

Eukaryotic cell lines

Policy information about cell lines

Cell line source(s)	All the cell lines were purchased from American Type Culture Collection (ATCC)
Authentication	each cell line has been authenticated by American Type Culture Collection
Mycoplasma contamination	All cell lines tested negative for mycoplasma contamination
Commonly misidentified lines (See ICLAC register)	*Name any commonly misidentified cell lines used in the study and provide a rationale for their use.*

Animals and other organisms

Policy information about studies involving animals; ARRIVE guidelines recommended for reporting animal research

Laboratory animals	4-week-old male BALB/c nude mice obtained from Experimental Animal Centre of Shanghai laboratory animal center were used in our study.
Wild animals	The study did not involve wild animals.
Field-collected samples	The study did not involve sample collected from the field.
Ethics oversight	All experimental procedures were approved by the Institutional Animal Care and Use Committee of Renji Hospital, School of Medicine, Shanghai Jiao Tong University.

Note that full information on the approval of the study protocol must also be provided in the manuscript.

ChIP-seq

Data deposition

☒ Confirm that both raw and final processed data have been deposited in a public database such as GEO.

☒ Confirm that you have deposited or provided access to graph files (e.g. BED files) for the called peaks.

Data access links<br>*May remain private before publication.*	https://www.ncbi.nlm.nih.gov/geo/query/acc.cgi?acc=GSE132887
Files in database submission	myc_peaks.bed<br>siRNA_myc_peaks.bed<br>myc_peaks.fastq.gz<br>siRNA_myc_peaks.fastq.gz
Genome browser session<br>(e.g. UCSC)	no session generated

Methodology

Replicates	NA
Sequencing depth	myc_peaks.fastq.gz: reads : 28363224; single end; length:150bp<br>siRNA_myc_peaks.fastq.gz:reads: 21363370; single end;length:150
Antibodies	c-Myc 13987 CST
Peak calling parameters	Peak calling was performed using MACS1.4 with the default parameters
Data quality	For all libraries, fastp/0.20 was used to assess quality of all libraries and no libraries were discarded due to low quality
Software	Genomic data analysis: bowtie/2.3.2; samtools/1.6;macs/1.4.1; R/3.4.2; Rstudio/1.2.1335;
